# Mitochondrial genome structure and composition in 70 fishes: a key resource for fisheries management in the South Atlantic

**DOI:** 10.1186/s12864-024-10035-5

**Published:** 2024-02-27

**Authors:** Marcela Alvarenga, Ananda Krishna Pereira D’Elia, Graciane Rocha, Clara Alvarez Arantes, Frederico Henning, Ana Tereza Ribeiro de Vasconcelos, Antonio Mateo Solé-Cava

**Affiliations:** 1 CENIMP, Centro Nacional para a Identificação Molecular do Pescado, Departamento de Genética, Instituto de Biologia, Universidade Federal do Rio de Janeiro (UFRJ), Rio de Janeiro, RJ 21941-590 Brasil; 2 LNCC, Laboratório Nacional de Computação Científica, Petrópolis, RJ 25651-076 Brasil; 3 BIOPOLIS Program in Genomics, Biodiversity and Land Planning, CIBIO - Centro de Investigação em Biodiversidade e Recursos Genéticos, InBIO Laboratório Associado, Campus de Vairão, Universidade do Porto, Vairão, 4485-661 Portugal

**Keywords:** Target-enrichment sequencing, Mitochondrial genome, Comparative genomics, Genome structure, Conservation genomics, Fisheries management

## Abstract

**Background:**

Phylogenetic gaps of public databases of reference sequences are a major obstacle for comparative genomics and management of marine resources, particularly in the Global South, where economically important fisheries and conservation flagship species often lack closely-related references. We applied target-enrichment to obtain complete mitochondrial genomes of marine ichthyofauna from the Brazilian coast selected based on economic significance, conservation status and lack of phylogenetically-close references. These included sardines (Dorosomatidae, Alosidae), mackerels (Scombridae) croakers (Sciaenidae), groupers (Epinephelidae) and snappers (Lutjanidae).

**Results:**

Custom baits were designed to enrich mitochondrial DNA across a broad phylogenetic range of fishes. Sequencing generated approximately 100k reads per sample, which were assembled in a total of 70 complete mitochondrial genomes and include fifty-two new additions to GenBank, including five species with no previous mitochondrial data. Departures from the typical gene content and order occurred in only three taxa and mostly involved tRNA gene duplications. Start-codons for all genes, except Cytochrome C Oxidase subunit I (*COI*), were consistently ATG, whilst a wide range of stop-codons deviated from the prevailing TAA. Phylogenetic analysis confirmed assembly accuracy and revealed signs of cryptic diversification within the *Mullus* genus. Lineage delimitation methods using *Sardinella aurita* and *S. brasiliensis* mitochondrial genomes support a single Operational Taxonomic Unit.

**Conclusions:**

Target enrichment was highly efficient, providing complete novel mitochondrial genomes with little sequencing effort. These sequences are deposited in public databases to enable subsequent studies in population genetics and adaptation of Latin American fish species and serve as a vital resource for conservation and management programs that rely on molecular data for species and genus-level identification.

**Supplementary Information:**

The online version contains supplementary material available at 10.1186/s12864-024-10035-5.

## Background

Research on mitochondrial genomes have provided comprehensive insights into molecular evolution patterns, population dynamics, and adaptive processes across a wide array of organisms [1–6]. Mitochondrial genomes have been pivotal in molecular evolution and global genetic barcoding initiatives for species identification due to its small size, high substitution rate [7], lack of recombination and large copy number [8, 9]. Mitochondrial sequence data has greatly improved management and conservation [10, 11], but its utility in the South Atlantic is restricted due to the lack of reference sequences for key species. High-throughput methods for obtaining complete mitochondrial genomes enhance this potential.

Molecular data is essential due to challenges in morphological identification arising from processing [12, 13]. While fish meat is commonly marketed as fillets and steaks [14, 15], economically relevant products like fish fingers, cod cake [16], surimi [17], canned tuna and sardines [18, 19] undergo extensive processing. This poses additional challenges enforcing labeling regulations, safeguarding endangered species and upholding of consumer rights [20]. Because variability in marker resolution across taxa demands the utilization of alternative regions or their combinations [21], complete mitochondrial genomes offer flexibility in primer design and enable refined taxa-specific molecular assays [22, 23] to address eco-evolutionary questions pertinent for management strategies [24, 25].

**Fig. 1:**
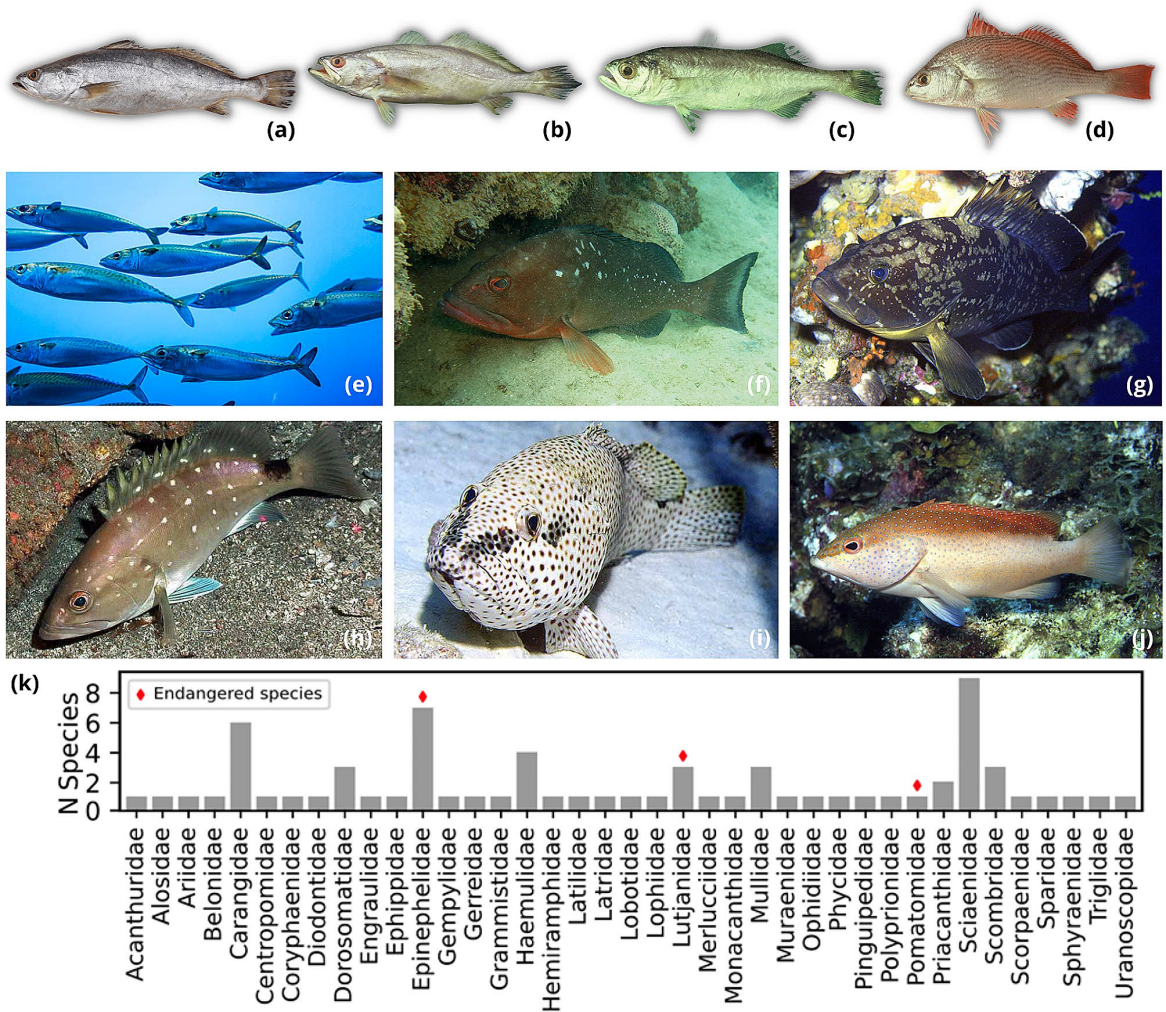
Species included in this study. Examples of emblematic species for conservation biology or economically relevant fisheries that lack reference mitochondrial genomes: croakers - (a) Cynoscion leiarchus, (b) Macrodon ancylodon, (c) Isopisthus parvipinnis, (d) Umbrina canosai; Brazilian sardine - (e) Sardinella brasiliensis; Serranids - (f) Epinephelus morio, (g) E. marginatus, (h) Hyporthodus niveatus, (i) E. adscensionis and (j) Cephalopholis fulva. (k) Total number of species included per family. Families containing at least one species evaluated as endangered in the IUCN RedList are indicated with a red diamowith. Photographies kindly provided by (a) Thiago Campos de SantanaElton Aviz, (b, c, d) Alfredo Carvalho Filho, (e) Costa Sul, (f) Claudio Sampaio, (g,i,j) Robert A. Patzner, (h) Smithsonian Tropical Research Institute.

While fish constitute the animal group with the highest number of sequenced mitochondrial genomes, only 9.7% of fish valid species are deposited in GenBank, for instance [26, 27]. Commercially important fish species from the Global South are particularly underrepresented, even though they frequently constitute valuable endemic fisheries or emblematic species of conservation concern. In fact, most of the highly valued fisheries from the South Atlantic lack mitochondrial genomes, including the Namorado perch (*Pseudopercis numida*), the Argentine hake (*Merluccius hubbsi*) and several croakers from the Scianidae family (*Cynoscion leiarchus, Macrodon ancylodon, Isopisthus parvipinnis, Umbrina canosai*, Fig. 1A-D). Sardines also hold significant economic value within Latin American fisheries. For instance, *Sardinella brasiliensis* (Fig. 1E) constituted nearly half of the fish discharges in Rio de Janeiro in the first semester of 2022, highlighting its substantial economic role as the most captured species in industrial fisheries [28]. Recently, a taxonomic revision merged *S. brasiliensis* into *S. aurita* with three other species: *S. lemuru, S. longiceps*, and *S. neglecta* [29]. However acceptance within the scientific community remains uncertain and this classification remains to be widely adopted [26], which hinders the delimitation of fish stocks and the sustainable exploitation of its natural populations [30].

Taxonomic uncertainty and limited genomic resources pose challenges to conservation efforts [30, 31]. Flagship species that are threatened but also marketable, such as the emblematic group of Epinephelidae comprising southern groupers (*Epinephelus morio, E. marginatus, Hyporthodus niveatus*, and *Cephalopholis fulva*, Fig. 1F-J), are notably affected by these limitations.

Here, we employed a target enrichment strategy using DNA hybridization capture to obtain 70 complete mitogenomes including some of the most important commercial species and flagship species for conservation programs in the western South Atlantic. We (a) fill important phylogenetic gaps for the molecular identification in fisheries conservation and management, (b) describe the main features and some rare departures form the typical mitochondrial genome, (c) use data from multiple mitochondrial genomes to assess the species status of economically important species.

## Materials and methods

### Sample selection

Species of teleost fishes were selected based on their economic importance, conservation status and phylogenetic distance to data that is currently available in sequence databases. The samples and tissues used were collected throughout the years by A.M.S.C through a collaborative effort (RENIMP, Portuguese acronym for National Network for the Molecular Identification of Fisheries) that also involved the morphological assignment by expert taxonomists. Seventy teleost species were included, representing 39 families and 15 orders (Fig. 1K; Table S1, Supporting Information).

### DNA extraction and library preparation

Genomic DNA was either extracted fresh from tissue (fin clips or muscle) preserved in ethanol at -20 °C or an archive of purified DNA. Fresh extracts were prepared from 20 mg tissue with DNeasy Blood and Tissue Kit (Qiagen), while archived DNA samples were originally extracted using various nucleic acid purification methods (salt extraction, Promega kit, phenol-chloroform). DNAs from both sources were screened using agarose gel electrophoresis (1%) and fluorometric quantification (Quantus dsDNA One, Promega). DNA samples were ascribed integrity scores ranging from 0 (completely degraded/absent) to 5 (high molecular weight) and included in the analysis if at least a faint band of genomic DNA was observed in addition to a smear. Genomic DNAs were maintained at -20 °C until use.

Sequencing libraries were prepared using the TruSeq Nano DNA HT Library Preparation Kit (Illumina) following the manufacturer’s protocol in a 96-well plate format. Library integrity was validated on the QIAxcel capillary electrophoresis system (Qiagen) using a high-resolution gel cartridge and the OM500 analysis method with 15 bp-3Kb alignment marker and a 100 bp-2.5 kb size/concentration standard (Fig. S1, Supporting Information) and were quantified with fluorometric assays (Quantus sDNA One, Promega or Qubit).

### Bait design and capture

A set of 25 taxa representing a large phylogenetic spectrum of fishes was retrieved from the National Center for Biotechnology Information (NCBI) sequence database (Table 1) and used to design custom RNA baits, following the MYBaits pipeline (Arbor Biosciences). The baits were designed with 80 nucleotides and 3x tiling density, with posterior collapsing baits within 95–99% of similarity, setting a total of 14,925 baits.

Sequencing libraries were enriched for mtDNA using the MYBaits Custom Kit with an 8-plex pooling strategy (Fig. S1, Supporting Information). Twelve capture pools of 100 to 500 ng in 7 µl (14 to 72 ng/µl) were obtained by combining sequencing libraries in equimolar amounts (Table S2, Supporting Information). Individual libraries were pooled by grouping samples in order to minimize within-pool variation in DNA integrity and concentration. Libraries were sequenced using an Illumina MiSeq sequencer with Reagent Micro kit v2 (Illumina).

**Table 1:** Reference sequences of GenBank used for the design of RNA probes used for targeted enrichment

Accession No.	Length (bp)	Species	Common Name	Family	Order	Class	Fishbase ID
KU244258	16 909	*Acanthurus nigrofuscus*	Brown surgeonfish	Acanturidae	Perciformes	Actinopterygii	4739
NC_011323	16 451	*Aluterus scriptus*	Scribbled leatherjacket filefish	Monacantidae	Tetraodontiformes	Actinopterygii	4275
NC_026307	17 243	*Anoxypristis cuspidate*	Pointed sawfish	Pristidae	Rhinopristiformes	Actinopterygii	8211
JQ728563	16 496	*Argyrosomus japonicus*	Japanese meagre	Scianidae	Perciformes	Actinopterygii	11979
NC_025942	16 750	*Atlantoraja castelnaui*	Spotback skate	Arhynchobatidae	Rajiformes	Chondrichthyes	14143
NC_003188	16 529	*Beryx splendens*	Splendid alfonsino	Berycidae	Beryciformes	Actinopterygii	1320
NC_024284	16 706	*Carcharhinus melanopterus*	Blacktip reef shark	Carcharhinidae	Carcharhiniformes	Chondrichthyes	877
NC_002761	18 705	*Conger myriaster*	Whitespotted conger	Congridae	Anguilliformes	Actinopterygii	302
NC_022509	17 017	*Epinephelus merra*	Honeycomb grouper	Epinephelidae	Perciformes	Actinopterygii	4923
NC_022691	16 701	*Isurus oxyrinchus*	Shortfin mako	Lamniformes	Lamnidae	Chondrichthyes	752
NC_005316	16 515	*Katsuwonus pelamis*	Skipjack tuna	Scombridae	Perciformes	Actinopterygii	107
NC_004380	16 479	*Lophius americanus*	American angler	Lophiidae	Lophiiformes	Actinopterygii	532
NC_016661	16 543	*Lutjanus argentimaculatus*	Mangrove red snapper	Lutjanidae	Perciformes	Actinopterygii	1407
FR751402	17 078	*Merluccius merluccius*	European hake	Merluccidae	Gadiformes	Actinopterygii	30
NC_003182	16 685	*Mugil cephalus*	Flathead grey mullet	Mugilidae	Mugiliformes	Actinopterygii	785
NC_002386	17 090	*Paralichthys olivaceus*	Bastard halibut	Paralichthyidae	Pleuronectiformes	Actinopterygii	1351
NC_002648	16 481	*Polymixia japonica*	Silver eye	Polymixiidae	Polymixiiformes	Actinopterygii	12996
KJ140136	16 780	*Rhinobatos schlegelii*	Brown guitarfish	Rhinobatidae	Rhinopristiformes	Actinopterygii	7969
NC_009587	16 651	*Sardinella maderensis*	Madeirian sardinella	Clupeidae	Clupeiformes	Actinopterygii	1047
NC_016867	16 500	*Sciaenops ocellatus*	Red drum	Scianidae	Perciformes	Actinopterygii	1191
NC_034748	16 734	*Squalus brevirostris*	Japanese shortnose spurdog	Squalidae	Squaliformes	Chondrichthyes	63317
NC_025328	16 690	*Squatina Formosa*	Taiwan angleshark	Squatinidae	Squatiniformes	Chondrichthyes	732
NC_026998	16 534	*Strongylura anastomella*	Pacific needlefish	Belonidae	Beloniformes	Actinopterygii	23039
NC_024026	16 558	*Trachinotus blochii*	Snubnose pompano	Carangidae	Perciformes	Actinopterygii	1963
NC_003173	16 065	*Zenopsis nebulosus*	Mirror dory	Zeidae	Zeiformes	Actinopterygii	3255

### Assembly and annotation

Preprocessing of reads, including quality trimming and demultiplexing was performed using BaseSpace (Illumina). Reads were assembled using NOVOPlasty, a software tool specifically designed for the rapid assembly of organelle genome [32]. A zebrafish complete mitochondrial genome sequence (NC_002333.2) was used as both reference and seed sequence, and the successful assemblies resulted in single circularized contigs. Some samples created a single contig successfully only when a sequence from a closely-related species was used as seed (see Table S3, Supporting Information). Samples which did not result in a single contig using NOVOPlasty were assembled in SPAdes [33] (Table S3, Supporting Information). To annotate the assembled mitochondrial genomes, we used MitoAnnotator, implemented in Mitofish*(*http://mitofish.aori.u-tokyo.ac.jp/), with posterior manual adjustment.

### Quality control and analysis

Quality control of the mitochondrial genomes encompassed several steps. Initially, assemblies with exceptionally low or high lengths were assessed for potential misassembly or chimeric sequences by comparison to closest related available mitogenome sequences’ length. Second, annotations from MitoAnnotator [34] were inspected to detect anomalies like stop codons within coding sequences, while also comparing tRNA, rRNA and CDS sizes with their nearest orthologs. Third, gene order deviations were investigated by analyzing the start position of all genes in each genome. Fourth, to validate assembly quality and target-capture sequence coverage, raw reads were aligned to assembled genome using the Burrows-Wheeler Aligner (BWA) [35] and SAMtools [36]. Pilon was used for assembly polishing by fixing nucleotide bases, rectifying mis-assemblies and filling sequence gaps [37]. Finally, we compared the size distributions between the genomes assembled in our study and those obtained from the GenBank RefSeq database for fish species.

In order to highlight the gain in resolution for molecular species identification, we retrieved sequences from the literature in which similarity to references was below 90% in protein coding genes. Although a similarity threshold varies between taxa, this level of divergence is not expected of orthologs from conspecifics or even congeneric species and is indicative of the lack of close reference sequences. These sequences were compared to our dataset using local BLAST searches and the level of similarity compared to public databases.

To inspect phylogenetic placement, unusual groupings and branch lengths, a maximum likelihood phylogenetic analysis was performed for the whole mitochondrial genomes in RAxML-NG v.1.0.3-master [38] using the GTR + G + I substitution model and 1000 bootstrap replicates. The best fitting substitution model was selected in MEGAx [39] with default settings. The analysis included all genomes assembled here along with their three closest-related sequences retrieved from GenBank and the reference sequences employed as probes. The final sequences matrix, which consisted of 267 complete mitochondrial sequences, was aligned using MAFFT v7.407 [40]. Since many of the species we sequenced lacked closely-related whole mitochondrial genomes available in public databases, we also conducted phylogenetic analysis using the five closest Cytochrome C Oxidase subunit 1 (*COI*) sequences from NCBI database, also by maximum likelihood in RAxML-NG v.1.0.3-master. In a singular instance for which the *COI* tree lacked sufficient phylogenetic resolution, an alternative phylogenetic analysis was conducted using another mitochondrial marker (*16 S rRNA*) based on a reduced dataset composed of sequences from the genus originally attributed to the unidentified sample (*Umbrina canosai*). For a detailed description of the quality control steps, see Supporting Information.

To test whether *Sardinella aurita* and *S. brasiliensis* form a single fish stock, two maximum likelihood phylogenetic inferences (based on *COI* and complete mitochondrial genome sequences) were performed in RAxML-NG v.1.0.3-master [38]. Species delimitation was performed within the *Sardinella* species, applying both the Automatic Barcode Gap Discovery [ABGD, 41] and Single-threshold generalized mixed Yule coalescent [GMYC; 42] to analyze *COI* and *12 S rRNA* sequences. For a detailed methodology of species delimitation, see the Supporting Information.

## Results

### Mitochondrial genome sequencing

#### Capture and assembly

We obtained 1.16 Gb of sequence data, representing 1.3x the expected output (0.89 Gb) of the MiSeq Reagent Kit v2 Micro. A total of 8,539,125 reads with ˜ 150 bp (7,378,870 passed pre-processing quality filtering, 97%) was achieved. Furthermore, the obtained data exhibited exceptional quality. This is especially notable since, despite encompassing degraded samples in our sample set as well, 84.12% of reads (*n* = 7,183,168) reached a quality score greater than 30 (QC > 30). The individual number of reads per sample ranged from 18.448 to 409.317 (Table S2, Supporting Information).

**Fig. 2:**
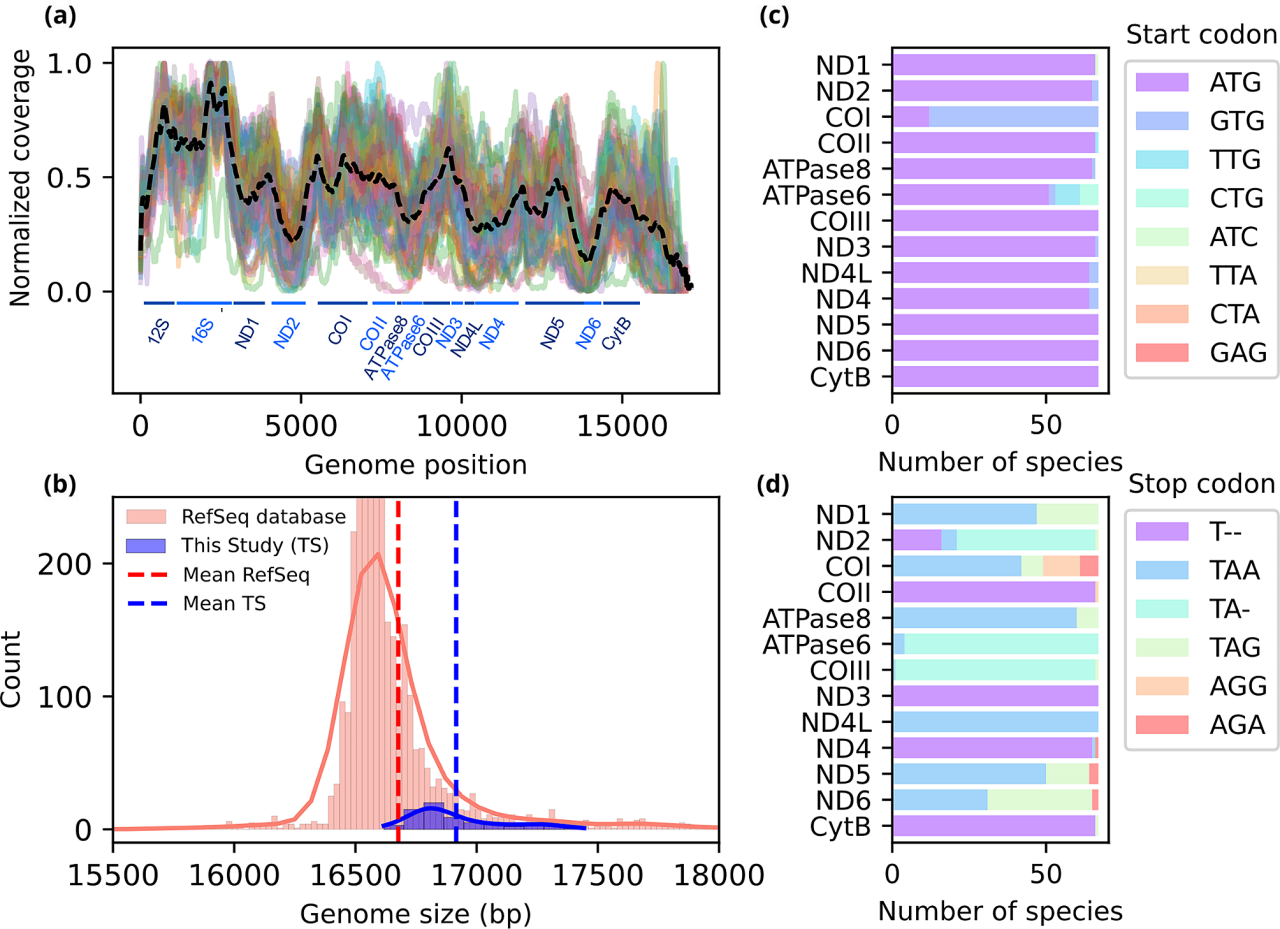
Structure of the sequenced mitochondrial genomes. (a) Normalized read coverage over the 15 mitochondrial genes. The genomic position of each gene is shown in the x-axis and the normalized coverage in the y-axis. The average coverage values are depicted by black lines with gaps; (b) count of reference genomes presenting different genome sizes in the RefSeq database (red) and in this study (blue). The mean genome size for each database is depicted by dashed lines. (c) Number of species containing each of the eight start codons (d) and six stop codons for the 13 protein coding gene

Sixty-five genomes were assembled using NOVOPlasty (61 as a circular genome) and five in SPAdes (genome in a single contig), with an average size of 16,916 bp (σ = 187; min: 16,617– max: 17,443). An average of 76% of reads were mapped to the assemblies, showcasing the efficiency of the enrichment protocol (σ = 13%; min: 33%– max: 97%), and an average mean reads coverage of 737x (σ = 604; min: 82– max: 3496) was obtained (Fig. 2A). Differences in coverage across different genes were consistent once comparing the mitochondrial genomes, with more conserved regions exhibiting the highest coverage, notably sections of the *16 S rRNA* gene (Fig. 2A). The Pilon pipeline for assembly polishing led to sequence corrections for 17 genomes, including replacement of bases in fifteen genomes and removal of bases in two genomes (Table S4, Supporting Information). Details of the assembly approach can be accessed in Table S3 of Supporting Information.

#### Features and organization of the newly assembled mitochondrial genomes

From the 70 mitochondrial genomes sequenced, 69 contained the full set of genes in the standard order: 13 protein coding genes (*NADH1*, *NADH2*, *COI*, *COII*, *ATPase 8*, *ATPase 6*, *COIII*, *NADH3*, *NADH4L*, *NADH4*, *NADH5*, *NADH6*, *CytB*), two rRNAs (*12 S rRNA* and *16 S rRNA*), 22 tRNAs (*tRNA-Phe, tRNA-Val, tRNA-Leu, tRNA-Ile, tRNA-Gln, tRNA-Met, tRNA-Trp, tRNA-Ala, tRNA-Asn, tRNA-Cys, tRNA-Tyr, tRNA-Ser, tRNA-Asp, tRNA-Lys, tRNA-Gly, tRNA-Arg, tRNA-His, tRNA-Ser, tRNA-Leu, tRNA-Glu, tRNA-Thr, tRNA-Pro*) and the D-loop control region. Eight tRNAs (*tRNA-Gln, tRNA-Ala, tRNA-Asn, tRNA-Cys, tRNA-Tyr, tRNA-Ser, tRNA-Glu*, and *tRNA-Pro*) and one protein-coding gene (*NADH6*) were encoded by the light (L) strand, and the remaining genes and tRNAs were encoded by the heavy (H) strand. Most mitochondrial genomes exhibited a conserved gene order (Fig. S2, Supporting Information). However, three genomes displayed variations: *Oligoplites saurus* had two *tRNA-Met*, *Pseudopercis numida* contained three *tRNA-Leu* and *Raneya brasiliensis* exhibited a distinctive deviation in gene order where the Cytochrome B (*CytB*) gene was encoded ahead of the NADH dehydrogenase subunit 6 (*NADH6*) gene.

Our main findings regarding the structure of the sequenced mitochondrial genomes were: (i) All the 70 genomes had all 13 protein coding genes; (ii) The complete reference mitochondrial genomes were 16,915 (σ = 188; min: 16,617– max: 17,443), as expected for fish mtDNA (Fig. 2B); (iii) The main start-codon was ATG for all genes except for *COI*, that presented a GTG start-codon in 82.6% of the genomes (Fig. 2C); (iv) More variation was observed in the stop-codons (Fig. 2D), with TAA being the most common one, although a majority of genes displayed alternative stop codons.

**Fig. 3:**
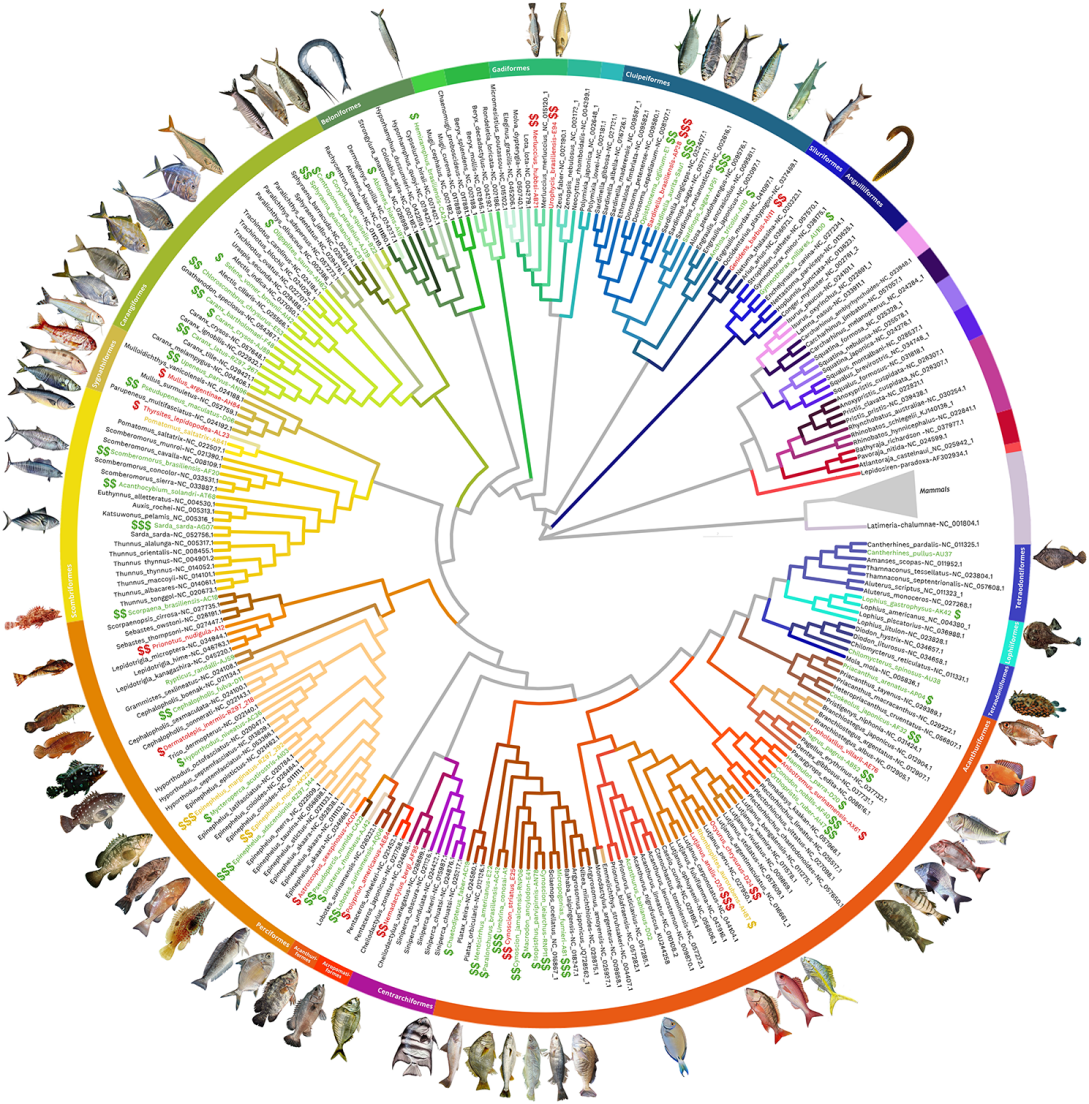
Complete mitochondrial genome phylogenetic tree. Sequences obtained here are highlighted in green, yellow or red for the IUCN categories of “Least Concern” “Vulnerable” “Data Deficient, Not Evaluated or Near Threatened”, respectively. The amount of dollar signs next to each sequence name are proportional to the commercial value of each species, with “$”, and “$” assigned to species with low, intermediate and high economic value, respectively. Families are indicated according to branch colors and orders are highlighted in bars. Non-monophyletic clades are highlighted in gray branches.

The 70 complete mitochondrial genomes obtained here represented species from 15 orders and 39 families. BLAST searches resulted in similarity values below 95% and 90% for 55 and 38 of our samples, respectively (Table S5, Supporting Information). Phylogenetic analysis of the complete mitochondrial genomes confirmed the filling of significant gaps on teleost tree reconstruction, yielding accurate species relationships (Fig. 3). The accurate phylogenetic placement of each species indicates that our sequences were correctly assembled. Sampled species were also accurately positioned in our phylogenetic reconstruction based on *COI* sequences (Fig. S3, Supporting Information). The phylogenetic placement of *Umbrina canosai* was confirmed by a *16 S rRNA* phylogenetic reconstruction, which provided enough taxonomic resolution for species-level identification (Fig. S4, Supporting Information). Moreover, the phylogenetic analysis of complete mitochondrial sequences supports the monophyly of 11 orders and 36 families assembled here (see Table S1, Supporting Information). The orders Perciformes, Tetraodontiformes and Acanthuriformes and the families Hemiramphidae and Belonidae were recovered as polyphyletic.

Our data also shed light on the debate regarding the taxonomy and evolution of the sardine species that occur on the Atlantic (*Sardinella aurita* and *S. brasiliensis*), proposed recently to be a single species (*S. aurita*) [29]. Despite the relatively high level of divergence observed in the whole mitochondrial genome phylogenetic tree (Fig. 3), lineage delimitation analyses did not recover *S. aurita* and *S. brasiliensis* as separate OTUs (Operational Taxonomic Units) (see “Sardine Lineage Delimitation Analyses” section on Supporting Information, which includes Fig. S5-S10 and Tables S7-S8). While the phylogenetic inference for *Sardinella COI* sequences did not indicate substantial genetic divergence between *S. aurita* and *S. brasilensis* (as depicted by branch lengths observed at Fig. S9, Supporting Information), the complete mitochondrial genome tree reconstruction based on *Sardinella* sequences did not present enough taxonomic resolution to enable species-level molecular identification due to low number of available mitogenomes (Fig. S10, Supporting Information). Our findings also imply potential cryptic diversity within the Mullidae family, as indicated by high levels of intraspecific genetic divergence in *Mullus argentinae* found in the *COI* gene phylogenetic analysis (Fig. S11, Supporting Information).

## Discussion

This study generated 70 complete mitochondrial genomes out of which 52 are novel for species of high commercial value, such as mackerel and anchovy, that prior to this work did not have any mitochondrial sequences in Genbank or BOLD System databases. We demonstrate the efficiency of target enrichment employing custom baits designed for broad phylogenetic coverage, in optimizing sequencing and analytical resources for mitochondrial genome sequencing. These resources supply vital backing for the delimitation of fishing stocks and for inspection of mislabeling and trade of endangered species, which are still predominant in many areas [43–46]. Proper regulation of fishing activity in areas for marine protection enhances conservation performance [47], and the tools provided here can be applied to evaluate the fishing market at the stocks level, to inspect the origin of exported seafood and the illegal sale of fish that inhabit protected areas.

### Molecular resources for fisheries management

Five of the 70 fish species analyzed are completely new to the NCBI database (*Astroscopus sexspinosus, Prionotus nudigula, Rypticus randalli, Anchoa tricolor and Thyrsites lepidopodea*) and three to the BOLD System database (*Anchoa tricolor*, *Thyrsites lepidopodea* and *Pseudopercis numida*). In total, 62 species lacked a complete record in RefSeq NCBI Database, 52 in the Nucleotide NCBI Database (complete, *n* = 52| partial, *n* = 5) and 62 in Mitofish. The new complete mitochondrial genomes that we deposited (*n* = 69) cover 38 families in 14 orders (Fig. 3), addressing important phylogenetic gaps revealed by similarity values below 95% in our BLAST analysis (Table S5, Supporting Information). The complete summary of species’ previous presence in public databases can be found in Table S6 of Supporting Information.

Groupers are subject to severe overfishing pressures [48], with around 10% of total species currently classified as threatened [49]. Here, we sequenced seven reference genomes from groupers of the Epinephelidae family, including the highly commercial Dusky grouper (*Epinephelus marginatus*), the Red grouper (*Ephinephelus morio*) and the Snowy grouper (*Hyporthodus niveatus*). Additionally, the marbled grouper (*Dermatolepis inermis*), which has insufficient data for the International Union for Conservation of Nature (IUCN) RedList analysis, also had its mitochondrial genome sequenced here.

Four other species sequenced here also do not have sufficient data for IUCN analysis: the commercial fishes Yellowtail snapper (*Ocyurus chrysurus*), the Wreckfish (*Polyprion americanus*), the Black margate (*Anisotremus surinamensis*) and the Brazilian sardinella (*Sardinella brasiliensis*). In addition, 10 species have not even been evaluated in the IUCN RedList (Not Evaluated, NE, in Table S1, Supporting Information), including the highly commercial Argentine hake (*Merluccius hubbsi*) and other relevant seafood for Latin-American economy, such as the Striped weakfish (*Cynoscion striatus*), the White sea catfish (*Genidens barbus*), the Castaneta (*Nemadactylus bergi*), the Red searobin (*Prionotus nudigula*) and the Brazilian codling (*Urophycis brasiliensis*).

Tunas and sardines (families Scombridae, Dorosomatidae and Alosidae) are extremely important for worldwide fisheries. Here, four sardines (*Opisthonema oglinum* and the highly commercial *Sardinella aurita, Sardinella brasiliensis* and *Sardinops sagax*) and three important representatives of the Scombridae family (*Sarda sarda*, *Scomberomorus brasiliensis* and *Acanthocybium solandri*) had their mitochondrial genomes sequenced. The two “Cavalas”, the Serra Spanish mackerel (*Scomberomorus brasiliensis*) and the Wahoo (*Acanthocybium solandri*), are often found as mislabeled tuna and can be added to canned tuna products [50–52].

Croakers and weakfishes, fish species from the Sciaenidae family, comprise a significant portion of the main demersal fishes exploited in South America [53–55]. We sequenced and analyzed nine Scianidae species, including the highly commercial Whitemouth croaker (*Micropogonias furnieri*) and the Argentine croaker (*Umbrina canosai*). One important addition to our dataset was the Smooth weakfish (*Cynoscion leiarchus*), one of the most relevant fishery resources in Brazil [56]. 

Two additional endangered species were included: the Bluefish (*Pomatomus saltatrix*) and the Vermilion snapper (*Rhomboplites aurorubens*). The Bluefish and the Vermilion snapper already had mitochondrial genomes available, but these lacked information on sampling locality. In total, for the 19 species with mitochondrial genomes available, 9 were present in the NCBI Reference Sequence Database (RefSeq), but only one includes information on geographical origin (*Caranx crysos* from Lybia). Providing genetic resources from a variety of geographic locations is important to enable the tracking of seafood point-of-origin [57].

Until now, the above-mentioned families had prominent gaps in molecular databases. The groupers (Epinephelidae) still lacked complete mitochondrial genomes for any Latin-American species, whilst croakers and weakfishes (Sciaenidae) only had three Latin-American species represented among the 79 mitochondrial genomes available for this family in GenBank. Notably, the *Cynoscion* and *Umbrina* genera, which encompasses the species with highest commercial relevance within Sciaenidae, had no complete mitochondrial genome yet available. Similarly, in sardines (Dorosomatidae and Alosidae), no mitogenomes were available for the only two Latin-American *Sardinella* species (*S. brasiliensis* and *S. aurita*). Furthermore, an entire family, Mullidae, did not have a mitochondrial genome for its Latin American representatives prior to this study. Consequently, this study provided new genomic resources for several underrepresented Latin American species and genera.

26% of the mitogenomes presented here belong to species on the RedList of IUCN. Providing more genomic resources to regulate those harming activities, especially for vulnerable fish species, are essential to enhance management efforts for the conservation of marine biodiversity from Latin America.

We have also sequenced species with data deficient for analysis by IUCN, which includes the Yellowtail snapper. The study and resource building for DD and NT species should be a conservation priority, since the DD status may represent an already threatened species without human knowledge, whilst NT represents species that are close to endangered categories. It has been shown that shark and ray species that were previously classified as DD and NT have now become threatened [58]. Another conservation gap that many Latin American fisheries resources face is the lack of assessment of species conservation status. Many of the species sequenced in this study were not even analyzed by IUCN, despite fishing pressures to which they are subjected. This is the case for the highly traded Argentine hake (*Merluccius hubbsi*), which is also one of the most consumed fishes in Brazil [59]. Providing more molecular resources for those species is a way to foment their study and assessments of their conservation status, leading to proper management actions.

### Capture sequencing as an efficient means of obtaining complete mitochondrial genomes

This study demonstrates the efficacy of the target-enrichment methodology in capturing a large breadth of fish diversity. We successfully obtained sequences from 70 fish species across a wide phylogenetic range. Even though some of our samples presented partially degraded DNA, we were able to successfully capture their genomes, which highlights the efficiency of the capture method in sequencing this type of sample. The capture methodology can be applied to assess highly processed seafood samples, such as cooked food, canned fish and fried mixtures (e.g. cod cake), covering important gaps in fisheries management.

The target capture sequencing methodology used in this study offers a cost advantage compared to other traditional techniques for sequencing mitochondrial genomes, such as long-range PCR, shotgun sequencing and Sanger sequencing [60–63]. We applied an 8-pool capture which, since excessive coverage was obtained for almost all of our samples, can be further multiplexed in future experiments. Successful applications with up to 96 pooling strategies and 8 samples per well plate have been reported [64–67]. Other cost-reduction techniques include reagents dilution, homebrew techniques as previously described [68]. Further studies analyzing cost reduction strategies for capture sequencing of fish samples [69] are desirable in order to further develop protocol for the widespread monitoring of fishing activities, including DNA barcoding [70].

### Organization of the mitochondrial genomes

Fish mtDNA is highly similar to the typical vertebrate mitochondrial genome, with 37 genes (13 protein-coding, 22 tRNA, and 2 rRNA genes) and 2 noncoding regions: Control Region (CR) and Origin of L-strand replication (OL) [71, 72]. These genes are oriented through two strands, L and H, where the first comprises the *NADH6* gene and 8 tRNAs, and the second all the rest [71, 73]. The mean base composition of protein-coding genes is A, 25.9%; C, 28.2%; G, 15.1%; and T, 30.8% (C ≈ A > T > G) in the H-strand of protein-coding genes, in which the Cytochrome C oxidase subunits and the Cytochrome B subunit are highly conserved, with emphasis on the Cytochrome C Oxidase subunit I (*COI*), with a high number of invariable sites between species. The rRNA genes (*12 S rRNA* and *16 S rRNA*) also have a huge amount of invariable sites, being even more conserved in fishes than in other vertebrate species [71]. Although most fish species exhibit the same gene order for mitochondrial genomes, variations in this order may occasionally occur [74]. The start codon is predominantly ATG, except in the *COI* gene where the GTG is the predominant start codon, and the stop-codons can be more variable, with four of the typical complete codons (TAA, TAG, AGA, and AGG) and three incomplete (TA-, T–, and AG-) [71].

Out of the 70 fish mitogenomes obtained, three had slight changes from the usual fish mitogenome bauplan: *Oligoplites saurus* (two *tRNA-Met*), *Pseudopercis numida* (three *tRNA-Leu*) and *Raneya brasiliensis* (inverted order of *NADH6* and *CytB*). The presence of repeats and duplications was recently indicated to be part of the vertebrate mitochondrial genome structure [75], and we retained these genomes. We opted against depositing one of the sequenced genomes (*Raneya brasiliensis*), due to its deviation from standard gene order regarding the *NADH6* and *CytB* genes. This deviation in *Raneya brasiliensis* was already reported before [76], and was attributed to gene rearrangements at the mitochondrial genomic region comprising the *NADH6* gene and D-loop. Despite this previous report supporting our findings, such phenomena should be validated by independent methods.

### Additional biological implications

Our phylogenetic analysis recovered 11 orders and 36 families as monophyletic (Fig. 3) and polyphyly for the orders Perciformes, Tetraodontiformes and Acanthuriformes and the families Hemiramphidae and Belonidae [26]. We did not consider the polyphyletic structure of these orders as a methodological error in our analysis, in light of previous research that have likewise highlighted their inability to recover monophyly or their controversial taxonomic status [77–81]. The monophyly of the families here recovered as polyphyletic, i.e. Hemiramphidae and Belonidae, has also been questioned before [82]. Conducting a comprehensive investigation into the phylogenetic relationships within these families and orders, utilizing a large genomic dataset and sample size that considers population variability, can be a fruitful avenue for future research.

Concerning the species status of *S. brasiliensis* and *S. aurita*, while branch lengths suggest a non-negligible level of divergence, lineage delimitation analysis (Supporting Information) failed to detect multiple Operational Taxonimic Units and support the merging of *S. aurita* and *S. brasiliensis* proposed by Stern et al. [29]. However, due to the large population sizes of sardines and the retention of ancestral polymorphisms, clarifying the phylogenetic relationship and taxonomy of the subgenus will require a larger set of loci, genomic approaches and broader geographic sampling. Currently, the *Sardinella Sardinella* subgenus comprises *S. aurita* (*S. aurita* + *S. brasiliensis*), *S. longiceps* (*S. longiceps* + *S. neglecta*) and *S. lemuru* [26]. Expanding geographic sampling and increasing sample size would not only enable the study of adaptation of *Sardinella aurita* to cold water, required to cross the Benguela barrier in southern Africa [83], but also complement the resources provided in this study. Furthermore, mitochondrial genomes have proven to be a valuable source for the study of adaptation genomics, particularly concerning metabolic responses [84–86].

On the other hand, our data also suggest the existence of hidden diversity within Mullidae. We found a relatively large degree of intraspecific divergence across a geographic range through the phylogenetic inference of the *COI* gene, which prompts a noteworthy taxonomic discourse within the Mullidae family, suggesting a possibly cryptic diversity in the species *Mullus argentinae.* This should be inspected in future studies using multiloci data.

## Conclusions

By filling significant phylogenetic gaps in sequence databases that have to date hindered molecular identification, this study presents valuable sequence resources for fisheries and conservation management in the South Atlantic. Our data also contribute to discussions concerning the classification and potential cryptic diversity in commercial species of sardines and *Mullus*. The success of targeted capture in accommodating samples with various degrees of input DNA fragmentation, as well as the sequencing and analytical efficiency that result from the enrichment, underscores its suitability for projects aiming at expanding the currently available complete mitochondrial genomes. The deposited sequences expand the potential of identification at species and population levels in the Atlantic South, ultimately contributing to sustainable exploitation and conservation of marine fishes and ecosystems.

### Electronic supplementary material

Below is the link to the electronic supplementary material.

Supplementary Material 1

## Data Availability

All genomes reported here were deposited in GenBank database under accession numbers PP032948-PP033016. Raw sequence data and scripts (UNIX and R) for all analyses performed here were deposited on Dryad (10.5061/dryad.rr4xgxdg4).
