# Supplementary material for: Mitochondrial genome structure and composition in 70 fishes: a key resource for fisheries management in the South Atlantic
Source: BMC Genomics. 2024 Feb 27;25:215. doi: 10.1186/s12864-024-10035-5 (PMC10898094; doi:10.1186/s12864-024-10035-5)
Supplement: Supplementary file 1 — Supplementary Material 1 [file 12864_2024_10035_MOESM1_ESM.pdf]

Supplementary Information

Samples 3
Supplementary Methods 6
Supplementary Tables 7
Supplementary Figures 18
Quality Control 20
Methodology 20
Results 21
Sardines lineage delimitation analysis 22
Outline 22
Methodology 22
ABGD 22
GMYC 22
Results & Discussion 23
ABGD Results 24
Lineages group per partitions 24
Third recursive partition - 12S 25
GMYC Results 28
Phylogenetic Implications 33
References 34

Figure S1. 0.7% Agarose gel showing the efficacy of the target-enrichment experiment, by comparing the DNA pools before and after the capture experiment with the MYBaits probes. 18
Figure S2. Variation in starting positions of 37 genes, including 13 protein-coding genes, 22 tRNA genes, 2 rRNA genes, and the D-loop region, across the 69 successfully assembled mitochondrial genomes. Although two genomes presented minor deviations in gene order (*Oligoplites saurus* with two tRNA-Met and *Pseudopercis numida* with three tRNA-Leu), the boxplot analysis revealed a pattern of variation across the different genes. This variation can be attributed to the differences in the evolutionary rate of different regions of the genome, which is expected due to differences in the selective pressures and functional constraints acting on these regions. 18
Figure S3. Phylogenetic inference based on the Cytochrome oxidase I (COI) sequences extracted from the mitochondrial genomes captured here, performed to confirm the identity of our mitochondrial genomes. Our sequences are highlighted in red and bootstrap values are displayed on each branch. 19
Figure S4. Phylogenetic inference of *Umbrina* and *Argyrosomus* 16S ribosomal RNA gene sequences obtained here (in red) and retrieved from GenBank database. The branch that supports the *Umbrina canosai* 16S rRNA sequence captured and the sequence retrieved form GenBank has zero-length, indicating nearly identical sequences. This analysis confirmed the identity of our *Umbrina canosai* sample (A1), which was not possible in our COI phylogeny. 20
Figure S5. Distance Histogram for *Sardinella* (a) CO1 and (b) 12S analyses. The x-axis represents the JC69 pairwise distance classes found between *Sardinella* sequences, and the y-axis represents the calculated

distance count (e.g. distance between Sardinella-aurita-Sau1 and Sardinella-brasiliensis-AP38 = 1 pairwise distance calculation). 24
Figure S6. Representation of the recursive and initial partitions for *Sardinella* (a) CO1 and (b) 12S analyses. The x-axis represents the maximum intraspecific divergence P calculated for the molecular operational taxonomic units delimited at each recursive partition (red) and initial partition (yellow), and the y-axis represents the number of groups (i.e. hypothetical species) delimited at each partition. 24
Figure S7. Summary of the GMYC species delimitation for (a) CO1 and (b) 12S sequences of *Sardinella*.... 28
Figure S8 Lineages delimited in the GMYC analyses of the (a) COI and (b) 12S genes. 29
Figure S9. Phylogenetic inference of the *Sardinella* Cytochrome oxidase I (COI) sequences available in GenBank database and the ones captured here, in red. The *Sardinella Sardinella* subgenus is highlighted in purple color.. 32
Figure S10. Phylogenetic inference of the Sardinella mitochondrial genomes available in GenBank database and the ones captured here, in red. The Sardinella Sardinella subgenus is highlighted with purple color........ 32
Figure S11. Phylogenetic inference of the *Mullus* Cytochrome oxidase I (COI) gene sequences available in GenBank and BOLD databases and the one captured here, in red. From the 7 sequences of the species *Mullus argentinae* available in BOLD database, 5 of them were obtained from samples collected in Argentina (FARG547-08, FARG545-08, FARG546-08, FARG548-08 and FARG549-08), and are clustered with the sequence captured here, obtained from a sample collected in southern Brazil (state of Santa Catarina). The other 2 BOLD sequences of *M. argentinae* (MFSP1895-11 and MFSP1984-11) were obtained from samples collected in southeast Brazil (state of São Paulo).. 34

SAMPLES

Table S1. Species sampled for this study with its locality and date.

Sample	Species	Common Name	Family	Subfamily	Order	Class	Commercial Relevance	IUCN	Sampling Date	Sampling Locality
AI97	*Ablennes hians*	Flat needlefish	Belonidae	NA	Beloniformes	Actinopterygii	Minor commercial	LC	24/02/2011	Santa Catarina, Brazil
AT68	*Acanthocybium solandri*	Wahoo	Scombridae	Scombrinae	Scombriformes	Actinopterygii	Commercial	LC	24/02/2013	Rio de Janeiro, Brazil
D02	*Acanthurus bahianus*	Barber surgeonfish	Acanthuridae	NA	Acanthuriformes	Actinopterygii	None	LC	20/11/2009	Pernambuco, Brazil
AI10	*Anchoa tricolor*	Piquitinga anchovy	Engraulidae	NA	Clupeiformes	Actinopterygii	Minor commercial	LC	07/02/2011	Rio de Janeiro, Brazil
AX61	*Anisotremus surinamensis*	Black margate	Haemulidae	Haemulinae	Acanthuriformes	Actinopterygii	Minor commercial	DD	17/09/2013	NA
AC02	*Astroscopus sexspinosus*	Brazilian stargazer	Uranoscopidae	NA	Perciformes	Actinopterygii	Minor commercial	NE	17/03/2011	Rio Grande do Sul, Brazil
AU37	*Cantherhines pullus*	Orangespotted filefish	Monacanthidae	NA	Tetraodontiformes	Actinopterygii	Subsistence	LC	09/06/2013	Rio de Janeiro, Brazil
F48	*Caranx barholomaei*	Yellow jack	Carangidae	NA	Carangiformes	Actinopterygii	Commercial	LC	12/12/2009	Ceará, Brazil
AJ89	*Caranx crysos*	Blue runner	Carangidae	NA	Carangiformes	Actinopterygii	Minor commercial	LC	21/01/2011	Bahia, Brazil
RZ97-267	*Caranx latus*	Horse-eye jack	Carangidae	NA	Carangiformes	Actinopterygii	Commercial	LC	01/01/1997	Bahia, Brazil
AJ39	*Centropomus parallelus*	Fat snook	Centropomidae	NA	Carangiformes	Actinopterygii	Minor commercial	LC	16/01/2011	Bahia, Brazil
D11	*Cephalopholis fulva*	Coney	Epinephelidae	NA	Perciformes	Actinopterygii	Commercial	LC	18/12/2009	Pernambuco, Brazil
AC19	*Chaetodipterus faber*	Atlantic spadefish	Ephippidae	NA	Acanthuriformes	Actinopterygii	Minor commercial	LC	17/03/2011	Rio Grande do Sul, Brazil
AU38	*Chilomycterus spinosus*	Brown burrfish	Diodontidae	NA	Tetraodontiformes	Actinopterygii	None	LC	09/06/2013	Rio de Janeiro, Brazil
E53	*Chloroscombrus chrysurus*	Atlantic bumper	Carangidae	NA	Carangiformes	Actinopterygii	Commercial	LC	28/02/2011	Rio Grande do Sul, Brazil
AF40	*Conodon nobilis*	Barred grunt	Haemulidae	Haemulinae	Acanthuriformes	Actinopterygii	Commercial	LC	17/03/2011	Rio Grande do Sul, Brazil
AF32	*Cookeolus japonicus*	Longfinned bullseye	Priacanthidae	NA	Acanthuriformes	Actinopterygii	Commercial	LC	15/03/2011	Rio Grande do Sul, Brazil
B22	*Coryphaena equiselis*	Pompano dolphinfish	Coryphaenidae	NA	Carangiformes	Actinopterygii	Minor commercial	LC	08/03/2010	Rio Grande do Norte, Brazil
RNP06	*Cynoscion jamaicensis*	Jamaica weakfish	Sciaenidae	NA	Acanthuriformes	Actinopterygii	Commercial	LC	05/12/2009	Rio de Janeiro, Brazil
RNP11	*Cynoscion leiarchus*	Smooth weakfish	Sciaenidae	NA	Acanthuriformes	Actinopterygii	Commercial	LC	05/12/2009	Rio de Janeiro, Brazil
E25	*Cynoscion striatus*	Striped weakfish	Sciaenidae	NA	Acanthuriformes	Actinopterygii	Commercial	NE	20/08/2010	Rio Grande do Sul, Brazil
RZ97-218	*Dermatolepis inermis*	Marbled grouper	Epinephelidae	NA	Perciformes	Actinopterygii	Minor commercial	DD	01/01/1997	Espirito Santo, Brazil
AJ42	*Diapterus rhombeus*	Caitipa mojarra	Gerreidae	NA	Acanthuriformes	Actinopterygii	Minor commercial	LC	16/01/2011	Bahia, Brazil
RZ97-144	*Epinephelus adscensionis*	Rock hind	Epinephelidae	NA	Perciformes	Actinopterygii	Highly Commercial	LC	01/01/1997	Espirito Santo, Brazil
RZ97-292	*Epinephelus marginatus*	Dusky grouper	Epinephelidae	NA	Perciformes	Actinopterygii	Highly Commercial	VU	01/01/1997	Espirito Santo, Brazil
AK22	*Epinephelus morio*	Red grouper	Epinephelidae	NA	Perciformes	Actinopterygii	Commercial	VU	20/08/2011	Bahia, Brazil
AN11	*Genidens barbus*	White sea catfish	Ariidae	Ariinae	Siluriformes	Actinopterygii	Commercial	NE	05/03/2011	Rio Grande do Sul, Brazil
AU100	*Gymnothorax miliaris*	Goldentail moray	Muraenidae	Muraeninae	Anguilliformes	Actinopterygii	Minor commercial	LC	NA	NA
D20	*Haemulon parra*	Sailor's grunt	Haemulidae	Haemulinae	Acanthuriformes	Actinopterygii	Minor commercial	LC	18/12/2009	Pernambuco, Brazil
CA24	*Hemiramphus brasiliensis*	Ballyhoo halfbeak	Hemiramphidae	NA	Beloniformes	Actinopterygii	Minor commercial	LC	12/11/2015	Rio de Janeiro, Brazil
AC36	*Hyporthodus niveatus*	Snowy grouper	Epinephelidae	NA	Perciformes	Actinopterygii	Commercial	VU	24/03/2011	Rio Grande do Sul, Brazil
AN78	*Isopisthus parvipinnis*	Bigtooth corvina	Sciaenidae	NA	Acanthuriformes	Actinopterygii	Minor commercial	LC	05/05/2011	Rio Grande do Sul, Brazil
AQ06	*Lobotes surinamensis*	Tripletail	Lobotidae	NA	Acanthuriformes	Actinopterygii	Commercial	LC	27/08/2012	São Paulo, Brazil
AK42	*Lophius gastrophysus*	Blackfin goosefish	Lophiidae	NA	Lophiiformes	Actinopterygii	Minor commercial	LC	11/04/2011	Rio de Janeiro, Brazil
AE76	*Lopholatilus villarii*	Tile fish	Latilidae	NA	Acanthuriformes	Actinopterygii	None	NE	04/03/2011	Rio Grande do Sul, Brazil

D30	*Lutjanus analis*	Mutton snapper	Lutjanidae	Lutjaninae	Acanthuriformes	Actinopterygii	Highly Commercial	NT	18/12/2009	Pernambuco, Brazil
E41	*Macrodon ancylodon*	King weakfish	Sciaenidae	NA	Acanthuriformes	Actinopterygii	Minor commercial	LC	20/08/2010	Rio Grande do Sul, Brazil
E32	*Menticirrhus americanus*	Southern kingcroaker	Sciaenidae	NA	Acanthuriformes	Actinopterygii	Commercial	LC	20/08/2010	Rio Grande do Sul, Brazil
AB31	*Merluccius hubbsi*	Argentine hake	Merlucciidae	NA	Gadiformes	Actinopterygii	Highly Commercial	NE	22/11/2010	Rio Grande do Sul, Brazil
A081	*Micropogonias furnieri*	Whitemouth croaker	Sciaenidae	NA	Acanthuriformes	Actinopterygii	Highly Commercial	LC	20/08/2010	Rio Grande do Sul, Brazil
AH84	*Mullus argentinae*	Argentine goatfish	Mullidae	NA	Syngnathiformes	Actinopterygii	Minor commercial	NE	23/02/2011	Santa Catarina, Brazil
AI03	*Mycteroperca acutirostris*	Comb grouper	Epinephelidae	NA	Perciformes	Actinopterygii	Minor commercial	LC	31/01/2011	Rio de Janeiro, Brazil
AF95	*Nemadactylus bergi*	Castaneta	Latridae	NA	Centrarchiformes	Actinopterygii	Commercial	NE	23/03/2011	Rio Grande do Sul, Brazil
D24	*Ocyurus chrysurus*	Yellowtail snapper	Lutjanidae	Lutjaninae	Acanthuriformes	Actinopterygii	Commercial	DD	18/12/2009	Pernambuco, Brazil
AH62	*Oligoplites saurus*	Leatherjacket	Carangidae	NA	Carangiformes	Actinopterygii	Minor commercial	LC	23/02/2011	Santa Catarina, Brazil
E1	*Opisthonema oglinum*	Atlantic thread herring	Dorosomatidae	NA	Clupeiformes	Actinopterygii	Commercial	LC	12/08/2010	Santa Catarina, Brazil
AI47	*Orthopristis rubra*	Corocoro grunt	Haemulidae	NA	Acanthuriformes	Actinopterygii	Commercial	LC	24/02/2011	Santa Catarina, Brazil
AB52	*Pagrus pagrus*	Red porgy	Sparidae	NA	Acanthuriformes	Actinopterygii	Commercial	LC	07/11/2010	Rio Grande do Sul, Brazil
AC43	*Paralonchurus brasiliensis*	Banded croaker	Sciaenidae	NA	Acanthuriformes	Actinopterygii	Minor commercial	LC	24/03/2011	Rio Grande do Sul, Brazil
AE84	*Polyprion americanus*	Wreckfish	Polyprionidae	NA	Acropomatiformes	Actinopterygii	Minor commercial	DD	04/03/2011	Rio Grande do Sul, Brazil
AB41	*Pomatomus saltatrix*	Bluefish	Pomatomidae	NA	Scombriformes	Actinopterygii	Highly Commercial	VU	25/11/2010	Rio Grande do Sul, Brazil
AP04	*Priacanthus arenatus*	Atlantic bigeye	Priacanthidae	NA	Acanthuriformes	Actinopterygii	Minor commercial	LC	23/06/2012	Rio de Janeiro, Brazil
A12	*Prionotus nudigula*	Red searobin	Triglidae	NA	Perciformes	Actinopterygii	Commercial	NE	17/08/2010	Rio Grande do Sul, Brazil
CA22	*Pseudopercis numida*	Namorado sandperc	Pinguipedidae	NA	Perciformes	Actinopterygii	Commercial	LC	15/12/2015	Rio de Janeiro, Brazil
D06	*Pseudupeneus maculatus*	Spotted goatfish	Mullidae	NA	Syngnathiformes	Actinopterygii	Commercial	LC	16/09/2009	Pernambuco, Brazil
AI55	*Raneya brasiliensis*	Banded cusk eel	Ophidiidae	Ophidiinae	Ophidiiformes	Actinopterygii	Minor commercial	LC	24/02/2011	Santa Catarina, Brazil
AH87	*Rhomboplites aurorubens*	Vermilion snapper	Lutjanidae	Lutjaninae	Acanthuriformes	Actinopterygii	Minor commercial	VU	23/02/2011	Santa Catarina, Brazil
AJ59	*Rypticus randalli*	Plain soapfish	Grammistidae	NA	Perciformes	Actinopterygii	NA	LC	17/01/2011	Bahia, Brazil
AG07	*Sarda sarda*	Atlantic bonito	Scombridae	Scombrinae	Scombriformes	Actinopterygii	Highly Commercial	LC	NA	NA
Sau1	*Sardinella aurita*	Round sardinella	Dorosomatidae	NA	Clupeiformes	Actinopterygii	Highly Commercial	LC	11/11/2017	Mediterranean Sea, Spain
AP38	*Sardinella brasiliensis*	Brazilian sardinella	Dorosomatidae	NA	Clupeiformes	Actinopterygii	Highly Commercial	DD	NA	NA
AP51	*Sardinops sagax*	South American pilchard	Alosidae	NA	Clupeiformes	Actinopterygii	Highly Commercial	LC	NA	NA
AF20	*Scomberomorus brasiliensis*	Serra Spanish mackerel	Scombridae	Scombrinae	Scombriformes	Actinopterygii	Commercial	LC	14/03/2011	Santa Catarina, Brazil
AC18	*Scorpaena brasiliensis*	Barbfish	Scorpaenidae	Scorpaenina e	Perciformes	Actinopterygii	Commercial	LC	17/03/2011	Rio Grande do Sul, Brazil
AI42	*Selene vomer*	Lookdown	Carangidae	NA	Carangiformes	Actinopterygii	Minor commercial	LC	24/02/2011	Santa Catarina, Brazil
AC87	*Sphyraena guachancho*	Guachanche barracuda	Sphyraenidae	NA	Carangiformes	Actinopterygii	Commercial	LC	04/04/2011	Santa Catarina, Brazil
AL23	*Thyrsites lepidopodea*	White snake mackerel	Gempylidae	NA	Scombriformes	Actinopterygii	Minor commercial	NE	03/04/2012	Rio de Janeiro, Brazil
A001	*Umbrina canosai*	Argentine croaker	Sciaenidae	NA	Acanthuriformes	Actinopterygii	Highly Commercial	LC	17/08/2010	Rio Grande do Sul, Brazil
AN96	*Upeneus parvus*	Dwarf goatfish	Mullidae	NA	Syngnathiformes	Actinopterygii	Commercial	LC	23/08/2011	NA
E094	*Urophycis brasiliensis*	Brazilian codling	Phycidae	NA	Gadiformes	Actinopterygii	Commercial	NE	31/08/2010	Rio Grande do Sul, Brazil

SUPPLEMENTARY METHODS

Even though fishes are the group of animals with the major quantity of mitochondrial genomes in RefSeq GenBank database, the total amount represents only 9.7% of fish valid species [1, 2]. This information was checked by applying the filters "((mitochondrion[Filter]) AND fish[Organism]) AND srcdb_refseq[Properties]" in GenBank database, which returned 3552 species. There are 36.422 valid fish species according to [1].

Supplementary Tables

Table S2. Methodology summary containing DNA quality (concentration and integrity), library concentration and capture assessment (sequencing pools with respective i5/i7 index and sequence, and the resulting number reads).

		DNA quality			Capture					
**Sample**	**Species**	**DNA Concentration (ng/uL)**	**Integrity Score**	**Library Concentration (ng/uL)**	**Pool**	**i7-index**	**i5-index**	**i7 sequence**	**i5 sequence**	**n.reads**
AI97	*Ablennes hians*	50	3	26.8	8	D707	D507	CTGAAGCT	CAGGACGT	65675
AT68	*Acanthocybium solandri*	56	3	72	5	D711	D502	TCTCGCGC	ATAGAGGC	66919
D02	*Acanthurus bahianus*	12	4	14	4	D706	D503	GAATTCGT	CCTATCCT	110972
AI10	*Anchoa tricolor*	13	3	14	4	D706	D505	GAATTCGT	AGGCGAAG	34046
AX61	*Anisotremus surinamensis*	0.767	3	23	6	D705	D507	ATTCAGAA	CAGGACGT	121637
AC02	*Astroscopus sexspinosus*	15	4	23	3	D705	D508	ATTCAGAA	GTACTGAC	54768
AU37	*Cantherhines pullus*	37	4	28	9	D708	D501	TAATGCGC	TATAGCCT	57275
F48	*Caranx barholomaei*	6.2	4	7.7	3	D702	D508	TCCGGAGA	GTACTGAC	122284
AJ89	*Caranx crysos*	9.3	3	11	9	D704	D504	GAGATTCC	GGCTCTGA	153711
RZ97-267	*Caranx latus*	7.7	4	9.1	3	D703	D505	CGCTCATT	AGGCGAAG	82567
AJ39	*Centropomus parallelus*	23	5	29	6	D707	D505	CTGAAGCT	AGGCGAAG	30750
D11	*Cephalopholis fulva*	14	4	18	11	D705	D502	ATTCAGAA	ATAGAGGC	82112
AC19	*Chaetodipterus faber*	117	3	4.4	6	D701	D503	ATTACTCG	CCTATCCT	168298
AU38	*Chilomycterus spinosus*	45	5	46	1	D709	D508	CGGCTATG	GTACTGAC	43861
E53	*Chloroscombrus chrysurus*	7.2	4	6	12	D702	D507	TCCGGAGA	CAGGACGT	113428
AF40	*Conodon nobilis*	9.9	3	15	9	D706	D507	GAATTCGT	CAGGACGT	159382
AF32	*Cookeolus japonicus*	58	4	66	1	D710	D508	TCCGCGAA	GTACTGAC	40980
B22	*Coryphaena equiselis*	21	4	16	8	D705	D501	ATTCAGAA	TATAGCCT	69588
RNP06	*Cynoscion jamaicensis*	32	4	27	10	D708	D502	TAATGCGC	ATAGAGGC	73105
RNP11	*Cynoscion leiarchus*	127	4	83	12	D711	D505	TCTCGCGC	AGGCGAAG	149344
E25	*Cynoscion striatus*	55	3	18	7	D705	D504	ATTCAGAA	GGCTCTGA	146221
RZ97-218	*Dermatolepis inermis*	6.4	5	8.1	10	D703	D501	CGCTCATT	TATAGCCT	61521
AJ42	*Diapterus rhombeus*	47	5	61	8	D710	D504	TCCGCGAA	GGCTCTGA	40879
RZ97-144	*Epinephelus adscensionis*	14	5	20	10	D705	D503	ATTCAGAA	CCTATCCT	41344
RZ97-292	*Epinephelus marginatus*	12	4	9	12	D703	D507	CGCTCATT	CAGGACGT	44094
AK22	*Epinephelus morio*	10	3	13	1	D704	D507	GAGATTCC	CAGGACGT	48946
AN11	*Genidens barbus*	142	5	156	3	D712	D503	AGCGATAG	CCTATCCT	33712
AU100	*Gymnothorax miliaris*	53	3	62	6	D710	D507	TCCGCGAA	CAGGACGT	37806
D20	*Haemulon parra*	7.8	4	6.7	8	D703	D502	CGCTCATT	ATAGAGGC	184098
CA24	*Hemiramphus brasiliensis*	43	3	35	8	D709	D503	CGGCTATG	CCTATCCT	82870

AC36	*Hyporthodus niveatus*	24	3	38	8	D709	D502	CGGCTATG	ATAGAGGC	18448
AN78	*Isopisthus parvipinnis*	4.15	3	7.8	7	D703	D504	CGCTCATT	GGCTCTGA	105786
AQ06	*Lobotes surinamensis*	8.7	3	10	9	D704	D501	GAGATTCC	TATAGCCT	77290
AK42	*Lophius gastrophysus*	17	4	27	7	D707	D508	CTGAAGCT	GTACTGAC	116663
AE76	*Lopholatilus villarii*	58	3	67	1	D711	D501	TCTCGCGC	TATAGCCT	56365
D30	*Lutjanus analis*	10	5	11	9	D704	D505	GAGATTCC	AGGCGAAG	102653
E41	*Macrodon ancylodon*	8.1	3	10	12	D703	D508	CGCTCATT	GTACTGAC	111103
E32	*Menticirrhus americanus*	5.5	4	4.7	3	D702	D503	TCCGGAGA	CCTATCCT	235267
AB31	*Merluccius hubbsi*	4.53	3	5	5	D702	D501	TCCGGAGA	TATAGCCT	182399
A081	*Micropogonias furnieri*	01.09	5	2.86	9	D701	D501	ATTACTCG	TATAGCCT	153337
AH84	*Mullus argentinae*	2	3	18	10	D705	D505	ATTCAGAA	AGGCGAAG	114621
AI03	*Mycteroperca acutirostris*	6	4	6.1	2	D702	D506	TCCGGAGA	TAATCTTA	72974
AF95	*Nemadactylus bergi*	23	3	25	12	D707	D502	CTGAAGCT	ATAGAGGC	86823
D24	*Ocyurus chrysurus*	13	4	14	4	D706	D504	GAATTCGT	GGCTCTGA	101460
AH62	*Oligoplites saurus*	39	3	44	5	D709	D505	CGGCTATG	AGGCGAAG	67697
E1	*Opisthonema oglinum*	4	5	4.97	4	D701	D506	ATTACTCG	TAATCTTA	143279
AI47	*Orthopristis rubra*	57	4	80	1	D711	D503	TCTCGCGC	CCTATCCT	58812
AB52	*Pagrus pagrus*	4.71	3	3.75	2	D701	D502	ATTACTCG	ATAGAGGC	223683
AC43	*Paralonchurus brasiliensis*	18	3	22	4	D705	D506	ATTCAGAA	TAATCTTA	122395
AE84	*Polyprion americanus*	53	3	56	11	D710	D505	TCCGCGAA	AGGCGAAG	56184
AB41	*Pomatomus saltatrix*	12	4	11	2	D704	D508	GAGATTCC	GTACTGAC	166630
AP04	*Priacanthus arenatus*	287	5	274	7	D712	D507	AGCGATAG	CAGGACGT	50573
A12	*Prionotus nudigula*	4.82	5	5.9	8	D702	D504	TCCGGAGA	GGCTCTGA	71882
CA22	*Pseudopercis numida*	144	3	121	12	D712	D501	AGCGATAG	TATAGCCT	129016
D06	*Pseudupeneus maculatus*	9	4	11	3	D704	D502	GAGATTCC	ATAGAGGC	99448
AI55	*Raneya brasiliensis*	31	4	30	6	D708	D503	TAATGCGC	CCTATCCT	134727
AH87	*Rhomboplites aurorubens*	11	5	9.2	11	D704	D503	GAGATTCC	CCTATCCT	182046
AJ59	*Rypticus randalli*	29	5	33	1	D708	D504	TAATGCGC	GGCTCTGA	90310
AG07	*Sarda sarda*	3	4	11	10	D704	D506	GAGATTCC	TAATCTTA	74601
Sau1	*Sardinella aurita*	8.9	3	13	11	D706	D502	GAATTCGT	ATAGAGGC	384056
AP38	*Sardinella brasiliensis*	9	5	35	3	D708	D505	TAATGCGC	AGGCGAAG	409317
AP51	*Sardinops sagax*	24	3	29	11	D707	D506	CTGAAGCT	TAATCTTA	29254
AF20	*Scomberomorus brasiliensis*	164	3	155	6	D712	D504	AGCGATAG	GGCTCTGA	52271
AC18	*Scorpaena brasiliensis*	24	5	47	7	D710	D501	TCCGCGAA	TATAGCCT	44912
AI42	*Selene vomer*	27	4	22.6	4	D707	D504	CTGAAGCT	GGCTCTGA	30649
AC87	*Sphyraena guachancho*	190	5	147	2	D712	D505	AGCGATAG	AGGCGAAG	45165
AL23	*Thyrsites lepidopodea*	110	3	79	2	D711	D504	TCTCGCGC	GGCTCTGA	73085
A001	*Umbrina canosai*	3.96	4	5	10	D701	D505	ATTACTCG	AGGCGAAG	178457
AN96	*Upeneus parvus*	22	3	25	3	D707	D501	CTGAAGCT	TATAGCCT	138326
E094	*Urophycis brasiliensis*	4.62	3	6.3	7	D702	D505	TCCGGAGA	AGGCGAAG	69011

Table S3. Detailed assembling approach, showing the assembler utilized, the reference and seed sequences (when assembled with NOVOPlasty) and whether created a circularized contig or not.

Sample	Species	Assembler	Reference Sequence	Seed Sequence	circularized
AI97	*Ablennes hians*	NOVOPlasty	NC_002333.2	NC_002333.2	yes
AT68	*Acanthocybium solandri*	NOVOPlasty	NC_002333.2	NC_002333.2	yes
D02	*Acanthurus bahianus*	NOVOPlasty	NC_002333.2	NC_002333.2	yes
AI10	*Anchoa tricolor*	NOVOPlasty	NC_002333.2	NC_002333.2	yes
AX61	*Anisotremus surinamensis*	NOVOPlasty	NC_025517	NC_025517	yes
AC02	*Astroscopus sexspinosus*	SPAdes	na	na	na, single contig
AU37	*Cantherhines pullus*	NOVOPlasty	NC_002333.2	NC_002333.2	yes
F48	*Caranx barholomaei*	NOVOPlasty	NC_002333.2	NC_002333.2	yes
AJ89	*Caranx crysos*	NOVOPlasty	NC_004406	NC_004406	yes
RZ97-267	*Caranx latus*	NOVOPlasty	NC_002333.2	NC_002333.2	yes
AJ39	*Centropomus parallelus*	SPAdes	na	na	na, single contig
D11	*Cephalopholis fulva*	NOVOPlasty	NC_002333.2	NC_002333.2	yes
AC19	*Chaetodipterus faber*	NOVOPlasty	NC_024580	NC_024580	yes
AU38	*Chilomycterus spinosus*	NOVOPlasty	NC_002333.2	NC_002333.2	yes
E53	*Chloroscombrus chrysurus*	NOVOPlasty	NC_002333.2	NC_002333.2	yes
AF40	*Conodon nobilis*	NOVOPlasty	NC_002333.2	NC_002333.2	yes
AF32	*Cookeolus japonicus*	NOVOPlasty	NC_002333.2	NC_002333.2	yes
B22	*Coryphaena equiselis*	NOVOPlasty	AB355907.1	KR086819.1	no
RNP06	*Cynoscion jamaicensis*	NOVOPlasty	NC_002333.2	NC_002333.2	yes
RNP11	*Cynoscion leiarchus*	NOVOPlasty	NC_002333.2	NC_002333.2	yes
E25	*Cynoscion striatus*	NOVOPlasty	NC_002333.2	NC_002333.2	yes
RZ97-218	*Dermatolepis inermis*	NOVOPlasty	NC_002333.2	NC_002333.2	yes
AJ42	*Diapterus rhombeus*	NOVOPlasty	NC_002333.2	NC_002333.2	yes
RZ97-144	*Epinephelus adscensionis*	NOVOPlasty	NC_002333.2	NC_002333.2	yes
RZ97-292	*Epinephelus marginatus*	NOVOPlasty	NC_002333.2	NC_002333.2	yes
AK22	*Epinephelus morio*	NOVOPlasty	NC_002333.2	NC_002333.2	yes
AN11	*Genidens barbus*	NOVOPlasty	NC_002333.2	NC_002333.2	yes
AU100	*Gymnothorax miliaris*	NOVOPlasty	na	MF041691.1	yes
D20	*Haemulon parra*	NOVOPlasty	NC_002333.2	NC_002333.2	yes
CA24	*Hemiramphus brasiliensis*	NOVOPlasty	NC_002333.2	NC_002333.2	yes
AC36	*Hyporthodus niveatus*	NOVOPlasty	NC_013829	NC_013829	yes
AN78	*Isopisthus parvipinnis*	NOVOPlasty	NC_025937	NC_025937	yes
AQ06	*Lobotes surinamensis*	NOVOPlasty	AB355912.1	HQ573299.1	no
AK42	*Lophius gastrophysus*	NOVOPlasty	NC_002333.2	NC_002333.2	yes
AE76	*Lopholatilus villarii*	NOVOPlasty	NC_012907	NC_012907	yes
D30	*Lutjanus analis*	NOVOPlasty	NC_002333.2	NC_002333.2	yes
E41	*Macrodon ancylodon*	NOVOPlasty	NC_002333.2	NC_002333.2	yes
E32	*Menticirrhus americanus*	NOVOPlasty	NC_044717	NC_044717	yes
AB31	*Merluccius hubbsi*	NOVOPlasty	MT410897.1	HM421971.1	no
A081	*Micropogonias furnieri*	NOVOPlasty	NC_017610	NC_017610	yes
AH84	*Mullus argentinae*	NOVOPlasty	NC_002333.2	NC_002333.2	yes

AI03	*Mycteroperca acutirostris*	NOVOPlasty	NC_002333.2	NC_002333.2	yes
AF95	*Nemadactylus bergi*	NOVOPlasty	NC_002333.2	NC_002333.2	yes
D24	*Ocyurus chrysurus*	NOVOPlasty	NC_002333.2	NC_002333.2	yes
AH62	*Oligoplites saurus*	NOVOPlasty	KF356397.1	GU225649.1	no
E1	*Opisthonema oglinum*	NOVOPlasty	NC_002333.2	NC_002333.2	yes
AI47	*Orthopristis rubra*	NOVOPlasty	NC_002333.2	NC_002333.2	yes
AB52	*Pagrus pagrus*	NOVOPlasty	NC_005146	NC_005146	yes
AC43	*Paralonchurus brasiliensis*	NOVOPlasty	NC_002333.2	NC_002333.2	yes
AE84	*Polyprion americanus*	NOVOPlasty	NC_002333.2	NC_002333.2	yes
AB41	*Pomatomus saltatrix*	NOVOPlasty	NC_002333.2	NC_002333.2	yes
AP04	*Priacanthus arenatus*	NOVOPlasty	NC_002333.2	NC_002333.2	yes
A12	*Prionotus nudigula*	NOVOPlasty	NC_002333.2	NC_002333.2	yes
CA22	*Pseudopercis numida*	NOVOPlasty	NC_002333.2	NC_002333.2	yes
D06	*Pseudupeneus maculatus*	NOVOPlasty	NC_002333.2	NC_002333.2	yes
AI55	*Raneya brasiliensis*	SPAdes	na	na	na, single contig
AH87	*Rhomboplites aurorubens*	NOVOPlasty	NC_002333.2	NC_002333.2	yes
AJ59	*Rypticus randalli*	NOVOPlasty	NC_002333.2	NC_002333.2	yes
AG07	*Sarda sarda*	NOVOPlasty	NC_002333.2	NC_002333.2	yes
Sau1	*Sardinella aurita*	NOVOPlasty	NC_002333.2	NC_002333.2	yes
AP38	*Sardinella brasiliensis*	NOVOPlasty	NC_033407	NC_033407	yes
AP51	*Sardinops sagax*	NOVOPlasty	NC_002616	NC_002616	yes
AF20	*Scomberomorus brasiliensis*	NOVOPlasty	NC_002333.2	NC_002333.2	yes
AC18	*Scorpaena brasiliensis*	NOVOPlasty	NC_002333.2	NC_002333.2	yes
AI42	*Selene vomer*	NOVOPlasty	NC_025566	NC_025566	yes
AC87	*Sphyraena guachancho*	SPAdes	na	na	na, single contig
AL23	*Thyrsites lepidopodea*	NOVOPlasty	NC_002333.2	NC_002333.2	yes
A001	*Umbrina canosai*	NOVOPlasty	NC_002333.2	NC_002333.2	yes
AN96	*Upeneus parvus*	NOVOPlasty	NC_025566	NC_025566	yes
E094	*Urophycis brasiliensis*	SPAdes	na	na	na, single contig

Table S4. Summary of corrections from Pilon pipeline.

Sample	Species	Changes	Position
AI10	*Anchoa tricolor*	replacement	4752; 4877, M > A / 4891, K > T
AU37	*Cantherhines pullus*	replacement	2213, R > A / 2220; 2227, M > C / 2225, R > G
F48	*Caranx barholomaei*	replacement	5015, K > G / 13898, H > T
B22	*Coryphaena equiselis*	replacement	no changes
218	*Dermatolepis inermis*	replacement	16384, M > A / 16391, W > A / 16395; 16398, Y > T / 16401, S > G/ 16404; 16413, C > A / 16409, C > T / 16421, R > G
AJ42	*Diapterus rhombeus*	replacement	13606, D > T / 13607, M > C / 16158, H > C
144	*Epinephelus adscensionis*	replacement	3502; 11241; 11650, K > G / 3507, K > T / 3770; 4976; 11573; 11685; 11698; 12707; 12780; 12785; 13213, M > C / 4542, D > T / 11378, S > C / 11572; 11700, W > T / 11654, S > G / 11655, M > A / 13134, Y > T
292	*Epinephelus marginatus*	replacement	14312, K > T / 14316, W > A
AU100	*Gymnothorax miliaris*	replacement	Adição 15454-(TCTAGCG)-15462
AC36	*Hyporthodus niveatus*	replacement	4495, W > A
AN78	*Isopisthus parvipinnis*	removal	1859
AH84	*Mullus argentinae*	replacement	4592; 10228; 10242, K > T / 4697, M > A / 5000; 5001, K > G / 5005, M > C
A12	*Prionotus nudigula*	replacement	4481; 4482, M > A / 4483, R > A / 4612, W > A / 4622, W > T / 8394, V > A / 8395; 8398, M > C / 8537, K > T
D06	*Pseudupeneus maculatus*	replacement	8703, M > C
AP51	*Sardinops sagax*	replacement	10508; 12238, M > A / 10515, W > A / 12295, M > C / 13954, R > A
AC18	*Scorpaena brasiliensis*	replacement	3528; 6089; 12983, K > G / 6098, W > A / 12643; 13439, R > A / 13045; 13738, M > C / 13742; 13745; 13841, M > A / 13752, K > T / 13851, B > G
AC87	*Sphyraena guachancho*	removal	16884-16889

Table S5. Similarity analysis on Basic Local Alignment Search Tool - nucleotide (BLASTn).

Sample	Species	Common Name	Query length	Subject length	subject.id.mitogenome	max.score	total.score	query.cover	E.value	Per.Ident	query.accession
AI97	*Ablennes hians*	Flat needlefish	16534	16825	*Ablennes hians mitochondrial DNA, complete genome*	20053	21921	99%	0	90.80%	AP006774.1
AT68	*Acanthocybium solandri*	Wahoo	16555	16556	Acanthocybium solandri mitochondrial DNA, complete sequence	29652	29652	100%	0	99%	AP012945.1
D02	*Acanthurus bahianus*	Barber surgeonfish	16480	17272	Ctenochaetus striatus mitochondrion, complete genome	19230	19546	96%	0	88.83%	KU244260.1
AI10	*Anchoa tricolor*	Piquitinga anchovy	16683	16675	Engraulis japonicus mitochondrial DNA, complete sequence	20087	20087	100%	0	88.43%	AP017957.1
AC02	*Astroscopus sexspinosus*	Brazilian stargazer	17157	16543	Lutjanus argentimaculatus mitochondrion, complete genome	5393	5393	42%	0	80.27%	JN182927.1
AU37	*Cantherhines pullus*	Orangespotted filefish	16427	16426	Cantherhines pardalis mitochondrial DNA, complete genome	29774	29774	100%	0	99.39%	AP009184.1
AJ89	*Caranx crysos*	Blue runner	16596	16595	Caranx crysos mitochondrion, complete genome	30081	30081	100%	0	99.39%	NC_057648.1
F48	*Caranx barholomaei*	Crevalle jack	16570	16595	Caranx crysos mitochondrion, complete genome	21004	21277	98%	0	90.74%	NC_057648.1
RZ97-267	*Caranx latus*	Bar jack	16598	16593	Caranx melampygus mitochondrial DNA, complete genome	28773	28773	100%	0	97.96%	AP004445.1
AJ39	*Centropomus parallelus*	Fat snook	16510	16514	Istiophorus albicans mitochondrial DNA, complete genome	4156	8582	77%	0	79.38%	AP006035.1
D11	*Cephalopholis fulva*	Coney	16642	16587	Cephalopholis sonnerati mitochondrion, complete genome	13140	20127	99%	0	87.66%	KC593378.1
AC19	*Chaetodipterus faber*	Atlantic spadefish	16344	16561	Platax teira mitochondrion, complete genome	17834	17834	96%	0	87.13%	KJ668153.1
AU38	*Chilomycterus spinosus*	Brown burrfish	16511	16519	Chilomycterus reticulatus mitochondrial DNA, complete genome	19019	19019	100%	0	87.47%	LC659947.1
E53	*Chloroscombrus chrysurus*	Atlantic bumper	16562	16593	Caranx melampygus mitochondrial DNA, complete genome	18547	18871	98%	0	87.85%	AP004445.1
AF40	*Conodon nobilis*	Barred grunt	16956	16785	Anisotremus scapularis mitochondrion, complete genome	13476	17591	93%	0	85.49%	MT259025.1
AF32	*Cookeolus japonicus*	Longfinned bullseye	16506	16506	Heteropriacanthus cruentatus mitochondrion, complete genome	28958	28958	100%	0	98.33%	NC_056807.1
B22	*Coryphaena equiselis*	Pompano dolphinfish	16659	1629	Coryphaena equiselis 16S ribosomal RNA , partial sequence, mitochondrion	2994	2994	9%	0	99.82%	FJ374811.1
RNP11	*Cynoscion leiarchus*	Acoupa weakfish	16493	16490	Nibea miichthioides mitochondrion, complete genome	19555	19555	99%	0	88.09%	KU738606.1
RNP06	*Cynoscion jamaicensis*	Jamaica weakfish	16502	16490	Nibea miichthioides mitochondrion, complete genome	20120	20120	99%	0	88.72%	KU738606.1
E25	*Cynoscion striatus*	Striped weakfish	16507	16490	Argyrosomus amoyensis mitochondrion, complete genome	14196	20594	99%	0	90.46%	KM257863.1
RZ97-218	*Dermatolepis inermis*	Marbled grouper	16561	16545	Hyporthodus octofasciatus mitochondrion, complete genome	18596	18873	98%	0	87.90%	JX135579.1
AJ42	*Diapterus rhombeus*	Irish mojarra	16566	16531	Prionurus laticlavius mitochondrion, complete genome	8131	10595	90%	0	80.96%	NC_057285.1
RZ97-144	*Epinephelus adscensionis*	Rock hind	16963	15702	Epinephelus akaara mitochondrial DNA, complete genome except for D-loop	11830	18862	92%	0	87.12%	AP005992.1

RZ97-292	*Epinephelus marginatus*	Dusky grouper	16984	16920	Epinephelus epistictus mitochondrion, complete genome	20687	21072	95%	0	90.44%	KC816460.1
AK22	*Epinephelus morio*	Red grouper	16756	15702	Epinephelus akaara mitochondrial DNA, complete genome except for D-loop	18620	18620	93%	0	88.09%	AP005992.1
AN11	*Genidens barbus*	White sea catfish	16703	16830	Sciades seemanni mitochondrial DNA, almost complete genome	21725	22210	100%	0	90.94%	AP012003.1
AU100	*Gymnothorax miliaris*	Goldentail moray	16374	16568	Gymnothorax meleagris mitochondrion, complete genome	22777	27000	99%	0	96.67%	MW151695.1
D20	*Haemulon parra*	Sailor's grunt	16820	16785	Anisotremus scapularis mitochondrion, complete genome	12844	17017	93%	0	84.65%	MT259025.1
CA24	*Hemiramphus brasiliensis*	Ballyhoo halfbeak	16546	16527	Cheilopogon spilonotopterus mitochondrion, complete genome	11588	14465	98%	0	84.21%	NC_039400.1
AC36	*Hyporthodus niveatus*	Snowy grouper	16556	16545	Hyporthodus octofasciatus mitochondrion, complete genome	25089	25089	100%	0	94.04%	JX135579.1
AN78	*Isopisthus parvipinnis*	Bigtooth corvina	16503	16490	Nibea miichthioides mitochondrion, complete genome	19536	19536	99%	0	88.07%	KU738606.1
AQ06	*Lobotes surinamensis*	Tripletail	16485	16782	Lobotes surinamensis mitochondrial DNA, complete genome	24797	24797	97%	0	94.57%	AB355912.1
AK42	*Lophius gastrophysus*	Blackfin goosefish	16469	16479	Lophius americanus mitochondrial DNA, complete genome	23824	23824	100%	0	92.775	AP004414.1
AE76	*Lopholatilus villarii*	Tile fish	16505	16540	Branchiostegus japonicus mitochondrial DNA, complete genome	16120	16526	98%	0	85.25%	AP006804.1
D30	*Lutjanus analis*	Mutton snapper	16505	16508	Lutjanus guttatus voucher Ecu 1 mitochondrion, complete genome	26264	26264	100%	0	95.39%	KT724723.1
E41	*Macrodon ancylodon*	King weakfish	16528	16490	Nibea miichthioides mitochondrion, complete genome	15934	16546	99%	0	85.08%	KU738606.1
E32	*Menticirrhus americanus*	Southern kingcroaker	16510	16499	Menticirrhus littoralis mitochondrion, complete genome	19189	19189	99%	0	87.70%	MT199157.1
AB31	*Merluccius hubbsi*	Argentine hake	16996	16853	Merluccius merluccius isolate DM435 mitochondrion	21760	22921	97%	0	91.84%	MT410897.1
A081	*Micropogonias furnieri*	Whitemouth croaker	16495	16496	Micropogonias furnieri mitochondrion, complete genome	30295	30295	100%	0	99.82%	MW646294.1
AH84	*Mullus argentinae*	Argentine goatfish	16606	16577	Mullus surmuletus isolate DM358 mitochondrion, complete genome	24646	24646	99%	0	93.52%	NC_052759.1
AI03	*Mycteroperca acutirostris*	Comb grouper	17156	16920	Epinephelus epistictus mitochondrion, complete genome	20666	20843	94%	0	90.42%	KC816460.1
AX61	*Anisotremus surinamensis*	Smalleye croaker	16845	16785	Anisotremus scapularis mitochondrion, complete genome	20722	20722	94%	0	90.13%	MT259025.1
AF95	*Nemadactylus bergi*	Castaneta	16652	16652	Cheilodactylus variegatus mitochondrion, complete genome	22810	22878	99%	0	91.61%	KP704218.1
D24	*Ocyurus chrysurus*	Yellowtail snapper	16501	16502	Lutjanus peru mitochondrion, complete genome	25320	25320	100%	0	94.37%	KR362299.1
AH62	*Oligoplites saurus*	Leatherjacket	17052	16767	Scomberoides lysan mitochondrion, complete genome	14122	14122	93%	0	82.76%	MZ329991.1
E1	*Opisthonema oglinum*	Atlantic thread herring	16714	16704	Dorosoma cepedianum isolate NEFC F17-103 mitochondrion, complete genome	18336	19406	99%	0	87.80%	MG570459.1
AI47	*Orthopristis rubra*	Corocoro grunt	17012	16785	Anisotremus scapularis mitochondrion, complete genome	12347	16291	92%	0	83.96%	MT259025.1

AB52	*Pagrus pagrus*	Red porgy	16687	16828	Pagellus erythrinus mitochondrion, complete genome	21778	21929	99%	0	90.29%	NC_037732.1
AC43	*Paralonchurus brasiliensis*	Banded croaker	16615	16500	Sciaenops ocellatus mitochondrion, complete genome	17213	17213	99%	0	85.61%	JQ286004.1
AE84	*Polyprion americanus*	Wreckfish	16515	16514	Stereolepis doederleini UU1901 mitochondrial DNA, complete genome	22020	22020	99%	0	90.76%	LC649807.1
AB41	*Pomatomus saltatrix*	Bluefish	16542	16544	Pomatomus saltatrix mitochondrial DNA, complete genome	28522	28522	100%	0	97.79%	AB355904.1
AP04	*Priacanthus arenatus*	Atlantic bigeye	16995	16866	Priacanthus tayenus mitochondrion, complete genome	9751	16906	96%	0	86.585	KX891352.1
A12	*Prionotus nudigula*	Red searobin	16599	16542	Naso lopezi mitochondrial DNA, complete genome	10176	10176	94%	0	78.58%	AP009163.1
CA22	*Pseudopercis numida*	Namorado sandperc	16791	16496	Siniperca knerii voucher QBSM201711005l mitochondrial, complete genome	6072	12708	96%	0	81.14%	MK430069.1
D06	*Pseudupeneus maculatus*	Spotted goatfish	16560	16749	Select seq NC_062603.1 Parupeneus heptacanthus isolate MuHK005 mitochondrial	8514	15569	99%	0	86.19%	NC_062603.1
AI55	*Raneya brasiliensis*	Banded cusk eel	17003	16520	Myripristis vittata mitochondrion, complete genome	1860	3793	36%	0	80.36%	MZ329989.1
AH87	*Rhomboplites aurorubens*	Vermilion snapper	16502	16502	Lutjanus peru mitochondrion, complete genome	23167	23167	100%	0	92.01%	KR362299.1
AJ59	*Rypticus randalli*	Plain soapfish	16515	16506	Grammistes sexlineatus mitochondrial DNA, complete genome	16561	16561	95%	0	85.70%	AP006794.1
AG07	*Sarda sarda*	Atlantic bonito	16503	16506	Sarda sarda isolate DM366 mitochondrion, complete genome	27309	27309	100%	0	96.54%	NC_052756.1
Sau1	*Sardinella aurita*	Round sardinella	16596	12475	Sardinella longiceps mitochondrion, partial genome	18004	21465	75%	0	98.20%	KJ956251.1
AP38	*Sardinella brasiliensis*	Brazilian sardinella	16595	12475	Sardinella longiceps mitochondrion, partial genome	17930	21397	75%	0	98.97%	KJ956251.1
AP51	*Sardinops sagax*	South American pilchard	16882	16883	Sardinops sagax mitochondrion, complete genome	29211	29211	100%	0	97.90%	NC_057117.1
AF20	*Scomberomorus brasiliensis*	Serra Spanish mackerel	16698	16705	Scomberomorus complete genome	26286	26286	99%	0	95.14%	KX925518.1
AC18	*Scorpaena brasiliensis*	Barbfish	16756	16972	Scorpaenopsis ramaraoi NMMBP:1280 mitochondrial DNA, complete genome	7125	12239	95%	0	82.61%	LC493915.1
AI42	*Selene vomer*	Lookdown	16558	16570	Alectis ciliaris mitochondrion, complete genome	19728	20365	99%	0	89.18%	KM522837.1
AC87	*Sphyraena guachancho*	Guachanche barracuda	16991	16699	Sphyraena jello voucher ECSFRI-BMY001 mitochondrial, complete genome	13103	17090	94%	0	84.93%	KT445895.1
AL23	*Thyrsites lepidopodea*	White snake mackerel	16571	16261	Caristius groenlandicus mitochondrial DNA, complete genome	16460	16707	97%	0	85.67%	AP012974.1
A001	*Umbrina canosai*	Argentine croaker	16495	16490	Nibea miichthioides mitochondrion, complete genome	19440	19440	99%	0	87.97%	KU738606.1
AN96	*Upeneus parvus*	Dwarf goatfish	16424	16563	Mulloidichthys vanicolensis mitochondrial DNA, complete genome	9295	14032	94%	0	82.46%	AP012310.1
E094	*Urophycis brasiliensis*	Brazilian codling	16823	15776	Phycis blennoides isolate DM422 mitochondrion, partial genome	11575	17113	91%	0	85.30%	MT410873.1

Table S6. Overall summary about the completeness of the current database, explaining our contribution to it.

		NCBI				Mitofish	BOLD	NCBI Reference Genome		
Sample	Species	Reference Genome	Complete Genome	Partial Genome	Genes	Complete Genome	COI gene	Accession Number	Paper	Geographic Range
AI97	*Ablennes hians*	yes	yes	no	yes - COI, 12S, ATPase6, ATPase8, CytB	yes	yes	AB373007	Setiamarga et al. 2008	not available
AT68	*Acanthocybium solandri*	yes	yes	no	yes - COI, 12S, CytB	yes	yes	AP012945	Iwasaki et al. 2013	not available
D02	*Acanthurus bahianus*	no	yes	no	yes - COI, CytB	no	yes	OP035278	Bemis et al. Unpublished	not available
AI10	*Anchoa tricolor*	no	no	no	No	no	no			
AX61	*Anisotremus surinamensis*	no	yes	no	yes - COI, CytB, ATPase8, 16S	no	yes	OP056909	Bemis et al. Unpublished	not available
AC02	*Astroscopus sexspinosus*	no	no	no	No	no	yes			
AU37	*Cantherhines pullus*	no	yes	no	yes - COI, 16S, 12S, CytB	no	yes	OP056845	Bemis et al. Unpublished	not available
F48	*Caranx barholomaei*	no	no	no	yes- COI, CytB, 12S, 16S	no	yes			
AJ89	*Caranx crysos*	yes	yes	no	yes - 12S, 16S, COI, ND5, CytB	yes	yes	MW435597	Jeon et al. Unpublished	Lybia
RZ97-267	*Caranx latus*	no	no	no	yes - COI, CytB, 16S	no	yes			
AJ39	*Centropomus parallelus*	no	no	no	yes - COI, CytB	no	yes			
D11	*Cephalopholis fulva*	no	no	no	yes - COI, ND4, 12S, 16S	no	yes			
AC19	*Chaetodipterus faber*	no	no	no	yes - COI, ND3, 16S, 12S	no	yes			
AU38	*Chilomycterus spinosus*	no	no	no	yes – COI	no	yes			
E53	*Chloroscombrus chrysurus*	no	yes	no	yes - COI, 16S, 12S, CytB	no	yes	OP035250	Bemis et al. Unpublished	not available
AF40	*Conodon nobilis*	no	no	no	yes - CtyB, ND4, COI, 16S	no	yes			
AF32	*Cookeolus japonicus*	no	no	no	yes - COI, 12S, 16S	no	yes			
B22	*Coryphaena equiselis*	no	yes	yes	yes - COI, CytB, ND2, 16S, 12S	no	yes	AB355907 MH576916	Miya et al. 2013; Xu et al. 2018	not available/China
RNP06	*Cynoscion jamaicensis*	no	no	no	yes - COI, ATPase6, ATPase8, CytB, 16S	no	yes			
RNP11	*Cynoscion leiarchus*	no	no	no	yes - COI, CytB, ATPase8, 16S	no	yes			
E25	*Cynoscion striatus*	no	no	no	yes - COI, ATPase6, ATPase8, 16S	no	yes			
RZ97-218	*Dermatolepis inermis*	no	no	no	yes - COI, CytB, 16S, 12S	no	yes			
AJ42	*Diapterus rhombeus*	no	no	no	yes - COI, 16S, 12S	no	yes			

RZ97-144	*Epinephelus adscensionis*	no	no	no	yes - COI, 12S, 16S	no	yes			
RZ97-292	*Epinephelus marginatus*	no	no	no	yes - CytB, 12S, ND4, 16S, COI	no	yes			
AK22	*Epinephelus morio*	no	no	no	yes - COI, 16S, 12S	no	yes			
AN11	*Genidens barbus*	no	no	no	yes - COI, ATPase8, ATPase6, 16S, 12S, CytB	no	yes			
AU100	*Gymnothorax miliaris*	no	no	no	yes - COI, CytB, 16S, 12S	no	yes			
D20	*Haemulon parra*	no	no	no	yes - COI, CytB, 16S	no	yes			
CA24	*Hemiramphus brasiliensis*	no	no	no	yes - CytB, 16S, COI	no	yes			
AC36	*Hyporthodus niveatus*	no	no	no	yes - COI, 12S, 16S	no	yes			
AN78	*Isopisthus parvipinnis*	no	no	no	yes - COI, CytB, 16S	no	yes			
AQ06	*Lobotes surinamensis*	yes	yes	no	yes - COI, 12S, CytB	yes	yes	AB355912	Satoh et al. 2016	not available
AK42	*Lophius gastrophysus*	no	no	no	yes - COI, CytB	no	yes			
AE76	*Lopholatilus villarii*	no	no	no	yes - COI	no	yes			
D30	*Lutjanus analis*	no	yes	no	yes - COI, CytB, 16S, 12S, ND4	no	yes	OP056852	Bemis et al. Unpublished	not available
E41	*Macrodon ancylodon*	no	no	no	yes - COI, CytB, 16S, ATPase6, ATPase8	no	yes			
E32	*Menticirrhus americanus*	no	yes	no	yes - COI, ND4, CytB, 16S, 12S	no	yes	OP057093	Bemis et al. Unpublished	not available
AB31	*Merluccius hubbsi*	no	no	no	yes - CytB, COI, 12S, 16S	no	yes			
A081	*Micropogonias furnieri*	no	yes	no	yes - COI, CytB, 16S, ATPase6, ATPase8	no	yes	MW646294	Kim et al. Unpublished	not available
AH84	*Mullus argentinae*	no	no	no	yes - COI	no	yes			
AI03	*Mycteroperca acutirostris*	no	no	no	yes - COI, 12S, 16S	no	yes			
AF95	*Nemadactylus bergi*	no	no	no	yes - COI, CtyB	no	yes			
D24	*Ocyurus chrysurus*	no	no	no	yes - ND4, CytB, COI, 16S	no	yes			
AH62	*Oligoplites saurus*	no	no	no	yes - COI, CytB, 16S	no	yes			
E1	*Opisthonema oglinum*	no	no	no	yes - COI, CytB, 16S, 12S	no	yes			
AI47	*Orthopristis rubra*	no	no	no	yes - COI, CytB, 16S	no	yes			
AB52	*Pagrus pagrus*	no	no	yes	yes - COI, CytB, 16S, 12S	no	yes			
AC43	*Paralonchurus brasiliensis*	no	no	no	yes - COI, CtyB, 16S	no	yes			
AE84	*Polyprion americanus*	no	no	no	yes - CytB, COI, 12S, 16S	no	yes			
AB41	*Pomatomus saltatrix*	yes	yes	no	yes - CytB, COI, 16S, 12S	yes	yes	AB355904	Miya et al. 2013	not available
AP04	*Priacanthus arenatus*	no	no	no	yes - COI, 12S, 16S, COII	no	yes			

A12	*Prionotus nudigula*	no	no	no	no	no	yes			
CA22	*Pseudopercis numida*	no	no	no	yes - COI	no	no			
D06	*Pseudupeneus maculatus*	no	yes	no	yes - COI, 12S, ATPase6, ATPase8, 16S	no	yes	OP056871	Bemis et al. Unpublished	not available
AI55	*Raneya brasiliensis*	no	yes	no	yes - COI	no	yes	LC341245	Fromm et al. 2019	Argentina
AH87	*Rhomboplites aurorubens*	no	no	no	yes - COI, CytB, ND4, 16S	no	yes			
AJ59	*Rypticus randalli*	no	no	no	no	no	yes			
AG07	*Sarda sarda*	yes	yes	no	yes - CytB. COI, 16S, 12S	yes	yes	MT410877	Margaryan, A. Unpublished	not available
Sau1	*Sardinella aurita*	no	no	no	yes - CytB, COI, 16S, 12S	no	yes			
AP38	*Sardinella brasiliensis*	no	no	no	yes - 16S, COI	no	yes			
AP51	*Sardinops sagax*	yes	yes	no	yes - 16s, CytB, COI, 12S	yes	yes	MW338734	Tang, F.; Chen, W. Unpublished	not available
AF20	*Scomberomorus brasiliensis*	no	no	no	yes - CytB, ND4, COI, ND2, ATPase8, ATPase6	no	yes			
AC18	*Scorpaena brasiliensis*	no	no	no	yes - 16S, COI	no	yes			
AI42	*Selene vomer*	no	yes	no	yes - 16S, COI, CytB	no	yes	OP035063	Bemis et al. Unpublished	not available
AC87	*Sphyraena guachancho*	no	no	no	yes - COI, 16S, 12S	no	yes			
AL23	*Thyrsites lepidopodea*	no	no	no	no	no	no			
A001	*Umbrina canosai*	no	no	no	yes - COI, 16S	no	yes			
AN96	*Upeneus parvus*	no	no	no	yes - COI	no	yes			
E094	*Urophycis brasiliensis*	no	no	no	yes - COI	no	yes			

Supplementary Figures

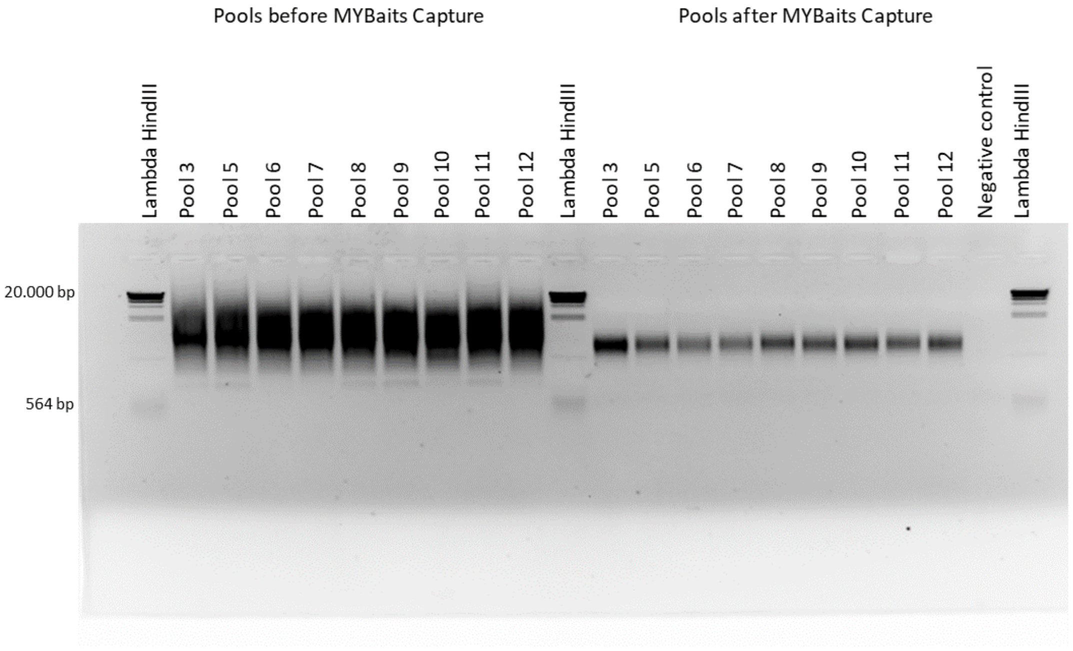

Figure S1. 0.7% Agarose gel showing the efficacy of the target-enrichment experiment, by comparing the DNA pools before and after the capture experiment with the MYBaits probes.

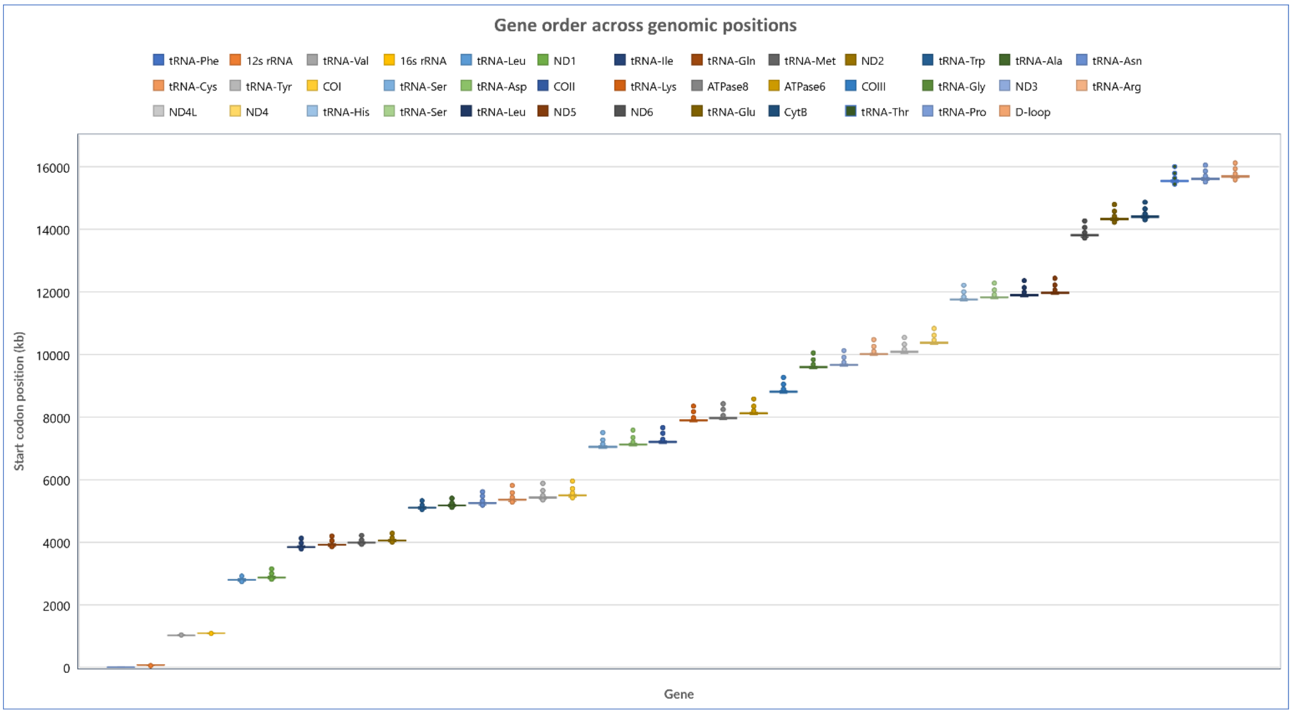

Figure S2. Variation in starting positions of 37 genes, including 13 protein-coding genes, 22 tRNA genes, 2 rRNA genes, and the D-loop region, across the 69 successfully assembled mitochondrial genomes. Although two genomes presented minor deviations in gene order (Oligoplites saurus with two tRNA-Met and Pseudopercis numida with three tRNA-Leu), the boxplot analysis revealed a pattern of variation across the different genes. This variation can be attributed to the differences in the evolutionary rate of different regions of the genome, which is expected due to differences in the selective pressures and functional constraints acting on these regions.

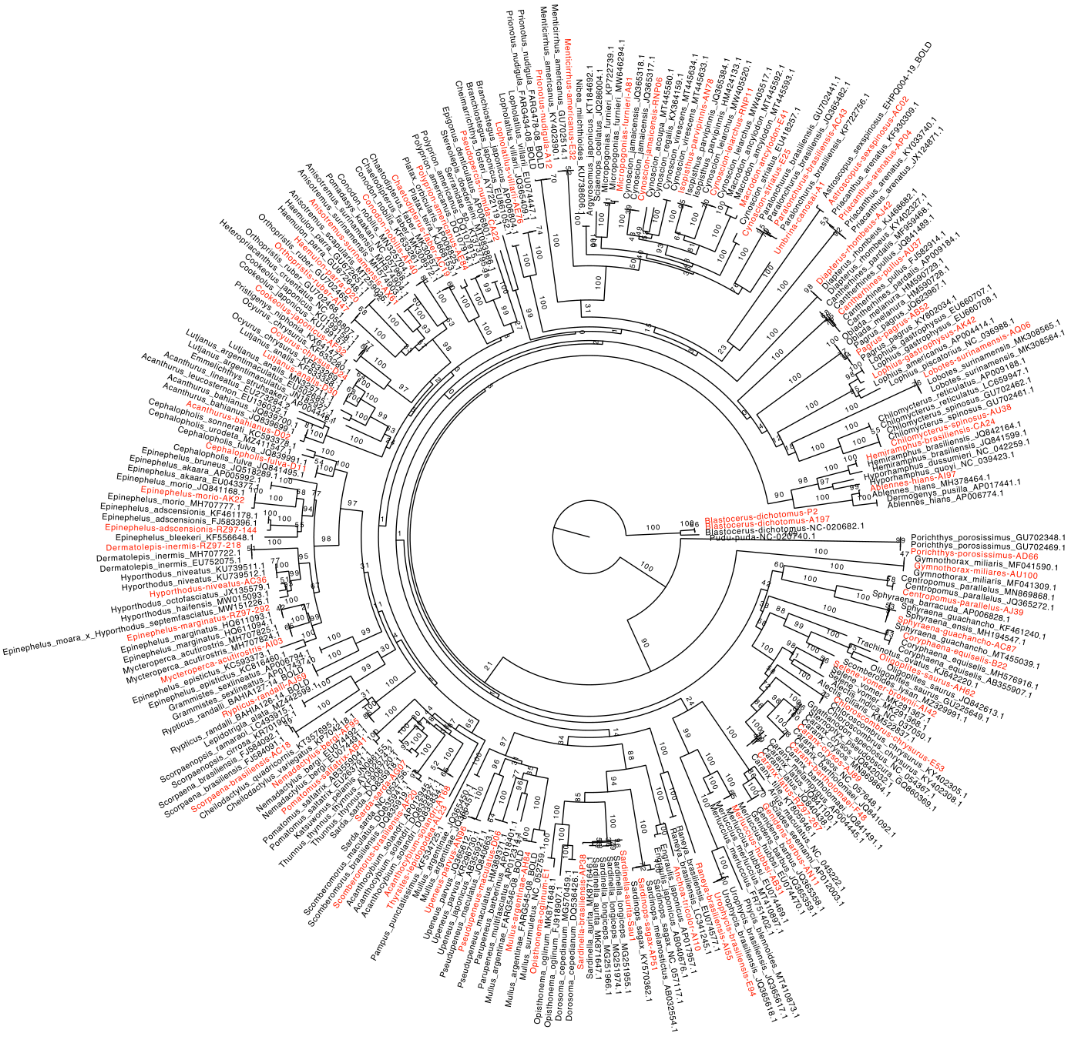

Figure S3. Phylogenetic inference based on the Cytochrome oxidase I (COI) sequences extracted from the mitochondrial genomes captured here, performed to confirm the identity of our mitochondrial genomes. Our sequences are highlighted in red and bootstrap values are displayed on each branch.

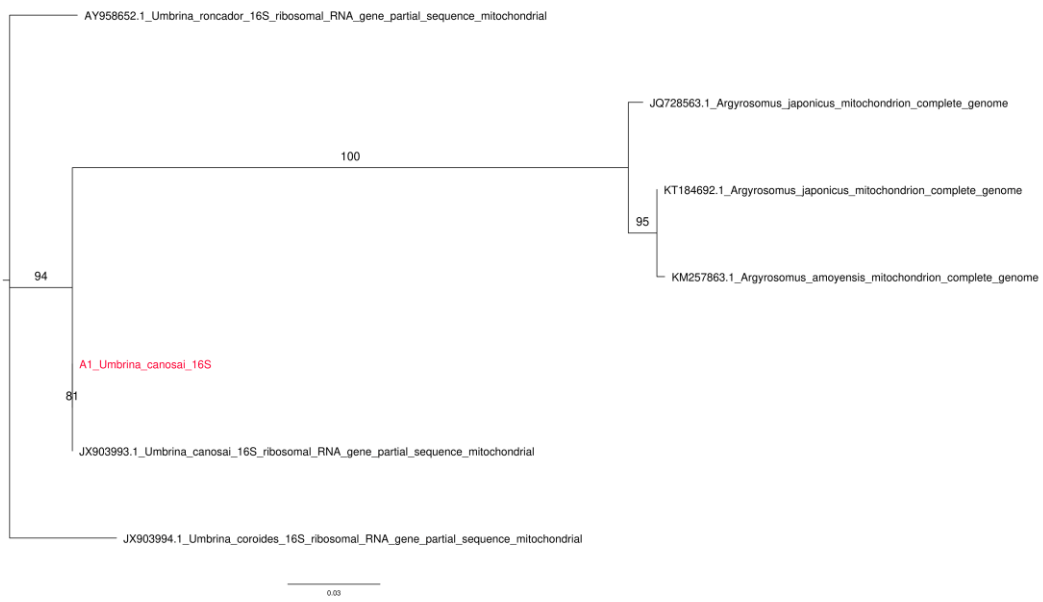

Figure S4. Phylogenetic inference of Umbrina and Argyrosomus 16S ribosomal RNA gene sequences obtained here (in red) and retrieved from GenBank database. The branch that supports the Umbrina canosai 16S rRNA sequence captured and the sequence retrieved form GenBank has zero-length, indicating nearly identical sequences. This analysis confirmed the identity of our Umbrina canosai sample (A1), which was not possible in our COI phylogeny.

QUALITY CONTROL

Methodology

The length distributions of the raw assemblies were analyzed. Assemblies with extremely low or high lengths were screened for indications of misassembly by comparison to the lengths of the closest-related available mitogenome (CRM) sequences. The CRMs were identified by blasting the whole mtDNA sequences and retrieving the top hit sorted by percentage identity and restricted to query cover > 80%. To check the annotation process, the annotated mitochondrial genomes were manually inspected. The size of each tRNA, rRNA and CDS were compared with their CRM. Also, the stop and the start codon of each CDS were first manually inspected in AliView v1.27 to verify other parameters of misassemble, such as truncations and duplications of protein-coding genes and the presence of stop-codons in the middle of sequences. We investigated possible deviations in gene order by analyzing the start position of all genes in each genome. To verify the quality and coverage of the target-capture sequencing, the raw reads were mapped to the assembled genome using Burrows-Wheeler Aligner (BWA) [3] and SAMTools [4]. To correct potential mismatches and other issues, we added the Pilon software in the mapping pipeline, a tool specially designed for assembling improvement [5].

To check the species identity of the mitochondrial genomes assembled here, a BLAST search was performed in the NCBI database using only the Cytochrome Oxidase subunit 1 (COI) sequences. The first hit was considered as the identity of the sample when with similarity >99%. A phylogenetic species identification was also performed by maximum likelihood for COI in RAxML-NG v.1.0.3-master [6] to confirm the phylogenetic placement of the samples using the monophyly as the criteria for species identification. The final sequence matrix consisted of 293 sequences and was aligned in MAFFT v7.407 (70 COI sequences from teleosts obtained here, 202 BLAST species hits, 17 COI sequences manually retrieved from GenBank and BOLD databases and 5 mammal sequences used as outgroups). COI sequences were manually retrieved from the GenBank and BOLD databases in cases where BLAST top hits did not match our query sequence species, even though sequences of the corresponding species were available in GenBank. The GTR+G+I was the nucleotide substitution model selected using MEGA X [7] according to the Bayesian information criterion [BIC; 8]. In cases where the COI tree did not have sufficient phylogenetic resolution, we developed phylogenies of other mitochondrial markers (Cytochrome b, Cytochrome Oxidase subunit II, 12s rRNA and 16s rRNA) for datasets containing only sequences of the genus initially assigned to the unidentified sample.

To inspect phylogenetic position, unusual groupings, and branch lengths a maximum likelihood phylogenetic analysis was performed for the whole mitochondrial genome in RAxML-NG v.1.0.3-master [6]. The final sequences matrix consisted of 267 complete mitochondrial sequences and was aligned in MAFFT v7.407 (69 sequences from teleost species obtained here that did not present any critical problems in other stages of the quality control and their top 3 BLAST species hits, the 25 complete mitochondrial genomes used to design the baits and their top 2 BLAST hits, 5 mammal sequences extracted from GenBank, one lungfish and one coelacanth). In order to remove alignment gaps, the automatic pipeline of trimAl v1.4.rev15 was applied. The GTR+G+I was the nucleotide substitution model selected according to the Bayesian information criterion (BIC; Schwarz, 1978) applied in the MEGA X software [7].

To investigate the relationship between *Sardinella aurita* and *S. brasiliensis*, two phylogenetic inferences by maximum likelihood were performed in RAxML-NG v.1.0.3-master [6]: 1) COI phylogeny including the species sequenced here and all *Sardinella* species available in GenBank and 2) mitogenome phylogeny including all mitochondrial genomes from *Sardinella* sp. available in GenBank and the ones produced here. An analysis of lineage delimitation was also performed within the *Sardinella* species group using the same dataset applied for the COI phylogenetic analysis and a second dataset including 12S sequences by applying two methods: 1) the Automatic Barcode Gap Discovery (ABGD; Puillandre et al. 2012). The ABGD method creates a genetic distance matrix (intra- and interspecific pair-wise distances) based on a pre-defined barcode gap that can split lineages into possible species. The analysis was performed in the web server (https://bioinfo.mnhn.fr/abi/public/abgd/abgdweb.html), using the Jukes-Cantor (JC69) as the nucleotide substitution model, with a relative gap width of 1.5 and intraspecific prior ranges from 0.001 to 0.1 in 10 steps. 2) Single-threshold generalized mixed Yule coalescent [GMYC; 9]. The GMYC method computes the maximum likelihood of models incorporating interspecific diversification and intraspecific clade occurrence on ultrametric time-tree. The analysis was performed with the R package splits v. 1.0-20 [SPecies' LImits by Threshold Statistics; 10] using the ultrametric tree produced in TreeAnnotator as the input file (see details below).

Results

All the 69 genomes had all 13 protein code genes in integrity. The main start-codon was the ATG for all genes except for COI, that has a GTG start-codon in 82,6% of the genomes obtained here (Figure 5a). A GTG start-codon was also found in other genes (except for CytB, COII, COIII, ND1 and ND5), but in minor quantity. ATPase6 was the second gene with less predominancy of ATG start-codon: 11 genomes had an ATPase6 starting with CTG, 2 with GTG and 1 with TTG. CytB also showed slight variation, with 5 genomes with a CTA start-codon, 3 TTA and 2 GTG. However, more variation was observed in the stop-codons (Figure 5b), with four complete stop-codons recorded (TAA, TAG, AGA, AGG – higher to lower quantity) and two incompletes (T-- and TA-). The presence of a complete stop-codon was more common in the genes COI, ATPase 8, ND1, ND4L, ND5 and ND6, with the stop-codon TAA at the end of all ND4L obtained here. The complete summary of gene annotation, size and stop- and start-codons can be found in tables S4, S7 and S8 in Supporting Information 1.

The BLAST search of the entire mtDNAs sequenced here retrieved the right congeneric species (Table S6). The complete reference mitochondrial genomes were 16,921 bp in average length (σ = 193; min: 16,617 – max: 17,337), the expected size for fish mtDNA (Figure 6). The average length of the CDSs and tRNAs separated is consistent with the fish average length as well. In addition, the quantity and size of protein coding (n = 13) genes, rRNA (n = 2) genes and tRNAs (n = 22) is similar to the typical teleost mitogenome (Tables S4 and S5, Supporting Information 1).

Similarity analysis and phylogenetic inference of the COI gene confirmed the identity and phylogenetic placement of all species that had their mtDNAs successfully sequenced but *Umbrina canosai*, which was confirmed with 16S (Figure S4), since the COI sequences from both GenBank and BOLD did not group with other *Umbrina* sp. sequences. We could not verify the species that do not have a sequence in GenBank or BOLD.

SARDINES LINEAGE DELIMITATION ANALYSIS

Outline

We collected samples of *Sardinella aurita* and *Sardinella brasiliensis* to contribute with the current discussion regarding the reclassification of the *Sardinella Sardinella* subgenus (*Sardinella aurita, S. brasiliensis, S. lemuru, S. longiceps* and *S. neglecta*). The analysis performed in the last main reclassification of the species N Stern, J Douek, M Goren and B Rinkevich [11] was made according to one nuclear gene (ribosomal 1st intro gene S7) and three fragments of mitochondrial genes: 671 bp of the Cytochrome Oxidase subunit 1 (COI), 458 bp of the Cytochrome B (Cytb), and 587 bp of the ribosomal 16S.

Methodology

One specimen of each species (*S. aurita* and *S. brasiliensis*) had their mitochondrial genome successfully sequenced in the target-enrichment capture. To proceed with the lineage delimitation analyses, we first performed a phylogenetic analysis of whole mitochondrial genome of *Sardinella* species in RAxML-NG v.1.0.3-master (Kozlov et al., 2019). Our dataset comprised the two mitochondrial genomes obtained here for *S. aurita* and *S. brasiliensis*, 17 *Sardinella* mitogenomes from GenBank database and the *Engraulis ringens* (MH732975.1) as the outgroup. The final matrix of 20 mitochondrial genomes was aligned in MAFFT v7.407. The GTR+G+I was the nucleotide substitution model selected, according to the Bayesian information criterion [BIC; 8] applied in the MEGA X [7].

We also extracted two mitochondrial genes to perform delimitation analysis: 1) COI, that has the largest database available for analysis, and 2) 12S, which is the gene not employed in Stern et al. 2018 that has more *Sardinella* species available at GenBank. We added here the *Sardinella brasiliensis* 12S sequence to the database to enable the lineage delimitation analysis in this gene.

The datasets comprised the two sequences extracted from our mitogenomes, species from GenBank (48 for COI and 38 for 12S) and the *Chirocentrus dorab* (AHY80612.1 for the COI tree and AP006229.1 for the 12S tree) as the outgroup comprising a total of 51 sequences for COI and 41 for 12S. A phylogenetic tree for the COI gene was first built in IQ-TREE [12]. For that, sequences were aligned in MAFFT v7.407. The HKY+G+I was selected as the nucleotide substitution model, according to the same methodology described above.

We also performed two methods for lineage delimitation analysis: 1) Automatic Barcode Gap Discovery [ABGD; 13] and 2) Single-threshold generalized mixed Yule coalescent [GMYC; 9].

ABGD

The ABGD model calculates a pairwise distance matrix, from which initial partitions are made from a maximum intraspecific distance P given by the barcode gap detected at each partition. From the initial partitions, the same barcode gap detection method is applied recursively, creating recursive partitions that tend to comprise a larger number of lineages. The initial partitions are usually more conservative (possibly overlumpers), and the recursive ones more generalist (possibly oversplitters). The analysis was performed in the web server (https://bioinfo.mnhn.fr/abi/public/abgd/abgdweb.html), using the Jukes-Cantor (JC69) as the nucleotide substitution model, with the standard parameters of relative gap (1.5) and intraspecific prior values (Pmin = 0.001, Pmax = 0.1, P steps = 10).

GMYC

To apply the GMYC method, we first built a phylogenetic tree by Bayesian inference using BEAST 2.6.7 [14]. To perform the phylogenetic inferences, we created NEXUS files corresponding to the alignment of the sequences in MEGA X [7] and then the XML format files in BEAUti 2.6.7 [14]. We applied the standard parameters for analysis, including the JC69 as nucleotide substitution model, the Strict Clock as molecular clock model and the Yule model as tree prior model. The output files provided by BEAST included files with the extension ".trees", which we used as the input file in TreeAnnotator 2.6.7 [14] to generate a single tree (the

maximum credibility tree - MCT) from the summary of information contained in trees sample set produced by BEAST. The Burnin' percentage parameter was set to 10%, the minimum posterior probability threshold for each node was kept at 0, and the node heights were set to the median heights option. The NEXUS-formatted MCT was applied for GMYC delimitation analysis. We conducted the GMYC analyses in the R package splits v. 1.0-20 [SPecies' LImits by Threshold Statistics; 10]. The delimitation model was implemented using the *gmyc* function, which has the single threshold method as its default setting.

Results & Discussion

The phylogenetic inference of the COI gene (Figure S9) of *Sardinella* species separated the subgenus *Sardinella Sardinella* into 5 groups according to their genetic distance, and the two specimens of *S. aurita* and *S. brasiliensis* sequenced here diverge in small branch sizes, possibly indicating little genetic distance. The phylogenetic tree of the complete mitochondrial genomes (Figure S10) did not show enough taxonomic resolution for species delimitation, since the number of available sequences was too small.

Based on the genetic distances calculated in the ABGD analysis of *Sardinella* sequences (Figure S5), the species of *Sardinella* analyzed with COI (n = 18) and 12S (n = 11) were split into 9 to 12 (COI) and 4 to 9 (12S) groups (Figure S6). The species *Sardinella aurita, S. brasiliensis, S. longiceps* and *S. lemuru* are grouped in all ABGD partitions with COI gene, even in the seventh recursive partition (probably an oversplitter). These results contribute to the hypothesis that the subgenus *Sardinella Sardinella* is one single cosmopolitan species (*S. aurita*). However, when we performed the analysis based on a matrix of sequences from a marker yet not evaluated before, the 12S gene, the subgenus *Sardinella Sardinella* has its species separated into two groups in all ABGD partitions: 1) *Sardinella aurita, S. brasiliensis* and *S. longiceps* and 2) *S. lemuru* and *S. zunasi*. The GMYC method has separated the *Sardinella* species into 10 lineages using COI sequences as input (Table S7) and 6 lineages using 12S rRNA sequences as input (Table S8), creating a single group for *Sardinella Sardinella* subgenus with COI gene and two groups with 12S gene, showing a consistent result between the two genes analyzed. Even though *S. lemuru* and *S. zunasi* share the same habitat in the Pacific Ocean, there is no record that these two species form a single clade. *S. brasiliensis* and *S. aurita* remained together in all delimitation analyses, which corroborates with the ongoing classification of *S. aurita* species (aurita + brasiliensis). However, *S. longiceps* also remained grouped with *S. aurita* and *S. brasiliensis*, which does not support the ongoing classification of *S. longiceps* as a single species (longiceps + neglecta). There were no *S. neglecta* sequences available for analysis. The overall results of the delimitation analysis point out a cosmopolitan *Sardinella aurita.* However, there are some inconsistencies within the analyses. We recommend new analyses applying a large set of specimens and molecular markers to clarify such incongruencies.

The remaining *Sardinella* species included in the analyses also demand an improved delimitation analysis with more samples and markers, as its results also highlighted some inconsistencies between genes, the current literature and the two delimitation models applied here [1, 15].

ABGD Results

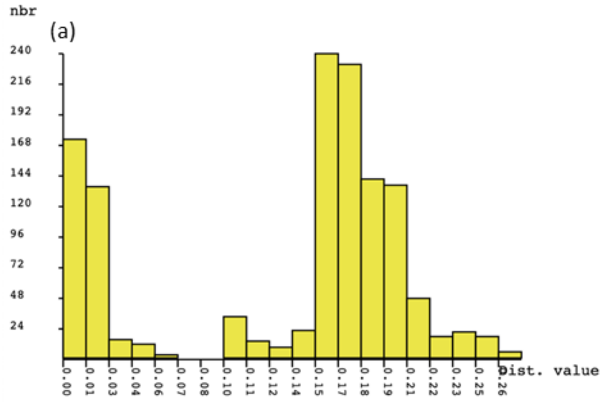

Figure S5. Distance Histogram for Sardinella (a) CO1 and (b) 12S analyses. The x-axis represents the JC69 pairwise distance classes found between Sardinella sequences, and the y-axis represents the calculated distance count (e.g. distance between Sardinella-aurita-Sau1 and Sardinella-brasiliensis-AP38 = 1 pairwise distance calculation).

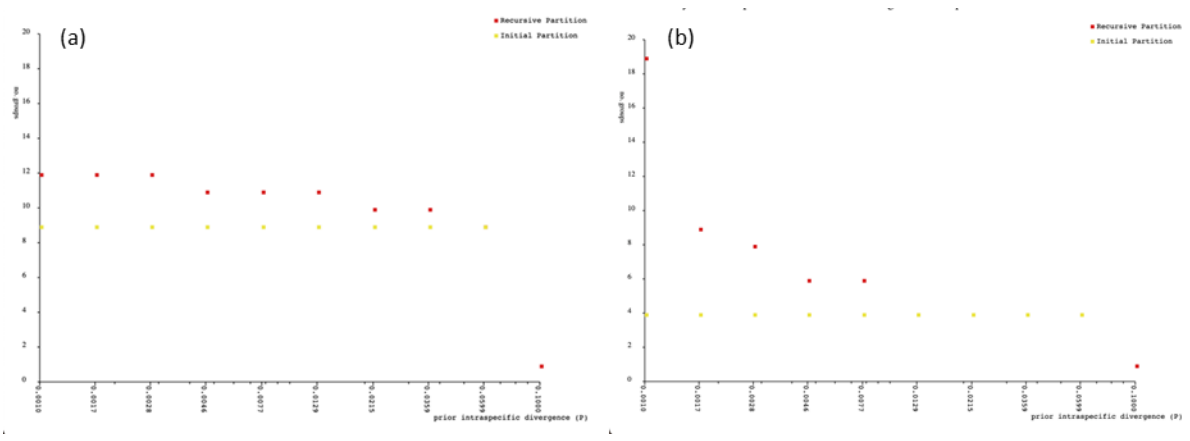

Figure S6. Representation of the recursive and initial partitions for Sardinella (a) CO1 and (b) 12S analyses. The x-axis represents the maximum intraspecific divergence P calculated for the molecular operational taxonomic units delimited at each recursive partition (red) and initial partition (yellow), and the y-axis represents the number of groups (i.e. hypothetical species) delimited at each partition.

Lineages group per partitions

Second recursive partition - 12S

Partition with prior maximal distance P=1.67e-03

Distance JC69 Jukes-Cantor MinSlope=1.500000

Group[1] n: 17 ;id: Sardinella_aurita_Sau1 Sardinella_brasiliensis_AP38 MG251941.1_Sardinella_longiceps KR000002.1_Sardinella_longiceps NC_033407.1_Sardinella_longiceps MG251937.1_Sardinella_longiceps MG251938.1_Sardinella_longiceps MG251939.1_Sardinella_longiceps MG251940.1_Sardinella_longiceps MG251942.1_Sardinella_longiceps MG251943.1_Sardinella_longiceps MG251944.1_Sardinella_longiceps LC708227.1_Sardinella_aurita LC708228.1_Sardinella_aurita LC708229.1_Sardinella_aurita EU552700.1_Sardinella_aurita DQ912032.1_Sardinella_aurita
Group[2] n: 7 ;id: KY964441.1_Sardinella_jussieu NC_035874.1_Sardinella_fijiensis MH011393.1_Sardinella_fijiensis MK400441.1_Sardinella_fijiensis NC_044472.1_Sardinella_fijiensis MH085733.1_Sardinella_jussieu MH085720.1_Sardinella_jussieu
Group[3] n: 3 ;id: MH995532.1_Sardinella_melanura MT753445.1_Sardinella_melanura

MH085627.1_Sardinella_melanura
Group[4] n: 5 ;id: MF536754.1_Sardinella_lemuru MF597091.1_Sardinella_lemuru NC_039553.1_Sardinella_lemuru MW118114.1_Sardinella_zunasi NC_057589.1_Sardinella_zunasi
Group[5] n: 2 ;id: KU665488.1_Sardinella_gibbosa NC_037131.1_Sardinella_gibbosa
Group[6] n: 2 ;id: AP011605.1_Sardinella_albella NC_016726.1_Sardinella_albella
Group[7] n: 2 ;id: AP009143.1_Sardinella_maderensis NC_009587.1_Sardinella_maderensis
Group[8] n: 1 ;id: MH085734.1_Sardinella_jussieu
Group[9] n: 1 ;id: MH085721.1_Sardinella_jussieu

Third recursive partition - 12S

Partition with prior maximal distance P=2.78e-03

Distance JC69 Jukes-Cantor MinSlope=1.500000

Group[1] n: 17 ;id: Sardinella_aurita_Sau1 Sardinella_brasiliensis_AP38 MG251941.1_Sardinella_longiceps KR000002.1_Sardinella_longiceps NC_033407.1_Sardinella_longiceps MG251937.1_Sardinella_longiceps MG251938.1_Sardinella_longiceps MG251939.1_Sardinella_longiceps MG251940.1_Sardinella_longiceps MG251942.1_Sardinella_longiceps MG251943.1_Sardinella_longiceps MG251944.1_Sardinella_longiceps LC708227.1_Sardinella_aurita LC708228.1_Sardinella_aurita LC708229.1_Sardinella_aurita EU552700.1_Sardinella_aurita DQ912032.1_Sardinella_aurita
Group[2] n: 7 ;id: KY964441.1_Sardinella_jussieu NC_035874.1_Sardinella_fijiensis MH011393.1_Sardinella_fijiensis MK400441.1_Sardinella_fijiensis NC_044472.1_Sardinella_fijiensis MH085733.1_Sardinella_jussieu MH085720.1_Sardinella_jussieu
Group[3] n: 3 ;id: MH995532.1_Sardinella_melanura MT753445.1_Sardinella_melanura MH085627.1_Sardinella_melanura
Group[4] n: 5 ;id: MF536754.1_Sardinella_lemuru MF597091.1_Sardinella_lemuru NC_039553.1_Sardinella_lemuru MW118114.1_Sardinella_zunasi NC_057589.1_Sardinella_zunasi
Group[5] n: 2 ;id: AP009143.1_Sardinella_maderensis NC_009587.1_Sardinella_maderensis
Group[6] n: 1 ;id: MH085734.1_Sardinella_jussieu
Group[7] n: 4 ;id: KU665488.1_Sardinella_gibbosa NC_037131.1_Sardinella_gibbosa AP011605.1_Sardinella_albella NC_016726.1_Sardinella_albella
Group[8] n: 1 ;id: MH085721.1_Sardinella_jussieu

Fourth recursive partition - 12S

Partition with prior maximal distance P=4.64e-03

Distance JC69 Jukes-Cantor MinSlope=1.500000

Group[1] n: 17 ;id: Sardinella_aurita_Sau1 Sardinella_brasiliensis_AP38 MG251941.1_Sardinella_longiceps KR000002.1_Sardinella_longiceps NC_033407.1_Sardinella_longiceps MG251937.1_Sardinella_longiceps MG251938.1_Sardinella_longiceps MG251939.1_Sardinella_longiceps MG251940.1_Sardinella_longiceps MG251942.1_Sardinella_longiceps MG251943.1_Sardinella_longiceps MG251944.1_Sardinella_longiceps LC708227.1_Sardinella_aurita LC708228.1_Sardinella_aurita LC708229.1_Sardinella_aurita EU552700.1_Sardinella_aurita DQ912032.1_Sardinella_aurita
Group[2] n: 12 ;id: KY964441.1_Sardinella_jussieu NC_035874.1_Sardinella_fijiensis KU665488.1_Sardinella_gibbosa NC_037131.1_Sardinella_gibbosa AP011605.1_Sardinella_albella NC_016726.1_Sardinella_albella MH011393.1_Sardinella_fijiensis MK400441.1_Sardinella_fijiensis NC_044472.1_Sardinella_fijiensis MH085733.1_Sardinella_jussieu MH085720.1_Sardinella_jussieu MH085721.1_Sardinella_jussieu
Group[3] n: 3 ;id: MH995532.1_Sardinella_melanura MT753445.1_Sardinella_melanura MH085627.1_Sardinella_melanura
Group[4] n: 5 ;id: MF536754.1_Sardinella_lemuru MF597091.1_Sardinella_lemuru

NC_039553.1_Sardinella_lemuru MW118114.1_Sardinella_zunas NC_057589.1_Sardinella_zunasi
Group[5] n: 2 ;id: AP009143.1_Sardinella_maderensis NC_009587.1_Sardinella_maderensis
Group[6] n: 1 ;id: MH085734.1_Sardinella_jussieu

Initial partitions - 12S

Initial Partition with prior maximal distance P=1.29e-02 ; Barcode gap distance = 0.078

Distance JC69 Jukes-Cantor MinSlope=1.500000

Group[1] n: 17 ;id: Sardinella_aurita_Sau1 Sardinella_brasiliensis_AP38 MG251941.1_Sardinella_longiceps KR000002.1_Sardinella_longiceps NC_033407.1_Sardinella_longiceps MG251937.1_Sardinella_longiceps MG251938.1_Sardinella_longiceps MG251939.1_Sardinella_longiceps MG251940.1_Sardinella_longiceps MG251942.1_Sardinella_longiceps MG251943.1_Sardinella_longiceps MG251944.1_Sardinella_longiceps LC708227.1_Sardinella_aurita LC708228.1_Sardinella_aurita LC708229.1_Sardinella_aurita EU552700.1_Sardinella_aurita DQ912032.1_Sardinella_aurita
Group[2] n: 15 ;id: KY964441.1_Sardinella_jussieu NC_035874.1_Sardinella_fijiensis KU665488.1_Sardinella_gibbosa NC_037131.1_Sardinella_gibbosa AP011605.1_Sardinella_albella NC_016726.1_Sardinella_albella AP009143.1_Sardinella_maderensis NC_009587.1_Sardinella_maderensis MH011393.1_Sardinella_fijiensis MK400441.1_Sardinella_fijiensis NC_044472.1_Sardinella_fijiensis MH085733.1_Sardinella_jussieu MH085720.1_Sardinella_jussieu MH085734.1_Sardinella_jussieu MH085721.1_Sardinella_jussieu
Group[3] n: 3 ;id: MH995532.1_Sardinella_melanura MT753445.1_Sardinella_melanura MH085627.1_Sardinella_melanura
Group[4] n: 5 ;id: MF536754.1_Sardinella_lemuru MF597091.1_Sardinella_lemuru NC_039553.1_Sardinella_lemuru MW118114.1_Sardinella_zunasi NC_057589.1_Sardinella_zunasi

First recursive partition – COI

Partition with prior maximal distance P=1.00e-03

Distance JC69 Jukes-Cantor MinSlope=1.500000

Group[1] n: 25 ;id: Sardinella-aurita-Sau1 Sardinella-brasiliensis-AP38 Sardinella-longiceps-AVI15835.1 Sardinella-longiceps-AVI16082.1 Sardinella-longiceps-AVI16030.1 Sardinella-longiceps-AVI16212.1 Sardinella-longiceps-AVI16303.1 Sardinella-aurita-QDB63769.1 Sardinella-aurita-AJG36172.1 Sardinella-aurita-ADJ36462.1 Sardinella-aurita-BCK59691.1 Sardinella-aurita-ARK08442.1 Sardinella-aurita-ALF62772.1 Sardinella-aurita-ARX77980.1 Sardinella-aurita-QWC70996.1 Sardinella-aurita-CAP45731.1 Sardinella-aurita-QIS89269.1 Sardinella-aurita-QIS89268.1 Sardinella-aurita-AKI88980.1 Sardinella-aurita-ARX77952.1 Sardinella-brasiliensis-CAP45732.1 Sardinella-lemuru-QIU80653.1 Sardinella-lemuru-QIU80654.1 Sardinella-lemuru-AVR48811.1 Sardinella-lemuru-ANN81745.1
Group[2] n: 2 ;id: Sardinella-jussieu-AGD80580.1 Sardinella-jussieu-QPT74108.1
Group[3] n: 2 ;id: Sardinella-maderensis-ARK08446.1 Sardinella-maderensis-YP_001293790.1
Group[4] n: 3 ;id: Sardinella-melanura-QNE86072.1 Sardinella-fimbriata-ADO15485.1 Sardinella-melanura-BBK60999.1
Group[5] n: 1 ;id: Sardinella-albella-YP_005089302.1
Group[6] n: 4 ;id: Sardinella-hualiensis-QQL03349.1 Sardinella-hualiensis-QQL03348.1 Sardinella-tawilis-ADO15497.1 Sardinella-tawilis-ADO15499.1
Group[7] n: 2 ;id: Sardinella-marquesensis-AIG24222.1 Sardinella-marquesensis-AIG24223.1
Group[8] n: 2 ;id: Sardinella-zunasi-ANN81739.1 Sardinella-zunasi-ANN81740.1
Group[9] n: 1 ;id: Chirocentrus-dorab-AHY80612.1
Group[10] n: 4 ;id: Sardinella-albella-UAX63140.1 Sardinella-fimbriata-ADK49636.1 Sardinella-goni-QNM64051.1 Sardinella-goni-QNM64052.1

Group[11] n: 4 ;id: Sardinella-gibbosa-QLM02870.1 Sardinella-gibbosa-QOR33582.1 Sardinella-sindensis-AZJ16169.1 Sardinella-sindensis-AZJ16172.1
Group[12] n: 1 ;id: Sardinella-jussieu-QIX12288.1

Third recursive partition – COI

Partition with prior maximal distance P=4.64e-03

Group[1] n: 25 ;id: Sardinella-aurita-Sau1 Sardinella-brasiliensis-AP38 Sardinella-longiceps-AVI15835.1 Sardinella-longiceps-AVI16082.1 Sardinella-longiceps-AVI16030.1 Sardinella-longiceps-AVI16212.1 Sardinella-longiceps-AVI16303.1 Sardinella-aurita-QDB63769.1 Sardinella-aurita-AJG36172.1 Sardinella-aurita-ADJ36462.1 Sardinella-aurita-BCK59691.1 Sardinella-aurita-ARK08442.1 Sardinella-aurita-ALF62772.1 Sardinella-aurita-ARX77980.1 Sardinella-aurita-QWC70996.1 Sardinella-aurita-CAP45731.1 Sardinella-aurita-QIS89269.1 Sardinella-aurita-QIS89268.1 Sardinella-aurita-AKI88980.1 Sardinella-aurita-ARX77952.1 Sardinella-brasiliensis-CAP45732.1 Sardinella-lemuru-QIU80653.1 Sardinella-lemuru-QIU80654.1 Sardinella-lemuru-AVR48811.1 Sardinella-lemuru-ANN81745.1
Group[2] n: 2 ;id: Sardinella-jussieu-AGD80580.1 Sardinella-jussieu-QPT74108.1
Group[3] n: 2 ;id: Sardinella-maderensis-ARK08446.1 Sardinella-maderensis-YP_001293790.1
Group[4] n: 3 ;id: Sardinella-melanura-QNE86072.1 Sardinella-fimbriata-ADO15485.1 Sardinella-melanura-BBK60999.1
Group[5] n: 5 ;id: Sardinella-albella-YP_005089302.1 Sardinella-gibbosa-QLM02870.1 Sardinella-gibbosa-QOR33582.1 Sardinella-sindensis-AZJ16169.1 Sardinella-sindensis-AZJ16172.1
Group[6] n: 4 ;id: Sardinella-hualiensis-QQL03349.1 Sardinella-hualiensis-QQL03348.1 Sardinella-tawilis-ADO15497.1 Sardinella-tawilis-ADO15499.1
Group[7] n: 2 ;id: Sardinella-marquesensis-AIG24222.1 Sardinella-marquesensis-AIG24223.1
Group[8] n: 2 ;id: Sardinella-zunasi-ANN81739.1 Sardinella-zunasi-ANN81740.1
Group[9] n: 1 ;id: Chirocentrus-dorab-AHY80612.1
Group[10] n: 4 ;id: Sardinella-albella-UAX63140.1 Sardinella-fimbriata-ADK49636.1 Sardinella-goni-QNM64051.1 Sardinella-goni-QNM64052.1
Group[11] n: 1 ;id: Sardinella-jussieu-QIX12288.1

Seventh recursive partition – COI

Partition with prior maximal distance P=2.15e-02

Distance JC69 Jukes-Cantor MinSlope=1.500000

Group[1] n: 25 ;id: Sardinella-aurita-Sau1 Sardinella-brasiliensis-AP38 Sardinella-longiceps-AVI15835.1 Sardinella-longiceps-AVI16082.1 Sardinella-longiceps-AVI16030.1 Sardinella-longiceps-AVI16212.1 Sardinella-longiceps-AVI16303.1 Sardinella-aurita-QDB63769.1 Sardinella-aurita-AJG36172.1 Sardinella-aurita-ADJ36462.1 Sardinella-aurita-BCK59691.1 Sardinella-aurita-ARK08442.1 Sardinella-aurita-ALF62772.1 Sardinella-aurita-ARX77980.1 Sardinella-aurita-QWC70996.1 Sardinella-aurita-CAP45731.1 Sardinella-aurita-QIS89269.1 Sardinella-aurita-QIS89268.1 Sardinella-aurita-AKI88980.1 Sardinella-aurita-ARX77952.1 Sardinella-brasiliensis-CAP45732.1 Sardinella-lemuru-QIU80653.1 Sardinella-lemuru-QIU80654.1 Sardinella-lemuru-AVR48811.1 Sardinella-lemuru-ANN81745.1
Group[2] n: 6 ;id: Sardinella-jussieu-AGD80580.1 Sardinella-albella-UAX63140.1 Sardinella-fimbriata-ADK49636.1 Sardinella-jussieu-QPT74108.1 Sardinella-goni-QNM64051.1 Sardinella-goni-QNM64052.1
Group[3] n: 2 ;id: Sardinella-maderensis-ARK08446.1 Sardinella-maderensis-YP_001293790.1
Group[4] n: 3 ;id: Sardinella-melanura-QNE86072.1 Sardinella-fimbriata-ADO15485.1 Sardinella-melanura-BBK60999.1
Group[5] n: 5 ;id: Sardinella-albella-YP_005089302.1 Sardinella-gibbosa-QLM02870.1 Sardinella-gibbosa-QOR33582.1 Sardinella-sindensis-AZJ16169.1 Sardinella-sindensis-AZJ16172.1
Group[6] n: 4 ;id: Sardinella-hualiensis-QQL03349.1 Sardinella-hualiensis-QQL03348.1 Sardinella-tawilis-ADO15497.1 Sardinella-tawilis-ADO15499.1
Group[7] n: 2 ;id: Sardinella-marquesensis-AIG24222.1 Sardinella-marquesensis-AIG24223.1
Group[8] n: 2 ;id: Sardinella-zunasi-ANN81739.1 Sardinella-zunasi-ANN81740.1
Group[9] n: 1 ;id: Chirocentrus-dorab-AHY80612.1

Group[10] n: 1 ;id: Sardinella-jussieu-QIX12288.1

Initital partitions – COI

Initial Partition with prior maximal distance P=1.00e-03 ; Barcode gap distance = 0.080

Distance JC69 Jukes-Cantor MinSlope=1.500000

Group[1] n: 25 ;id: Sardinella-aurita-Sau1 Sardinella-brasiliensis-AP38 Sardinella-longiceps-AVI15835.1 Sardinella-longiceps-AVI16082.1 Sardinella-longiceps-AVI16030.1 Sardinella-longiceps-AVI16212.1 Sardinella-longiceps-AVI16303.1 Sardinella-aurita-QDB63769.1 Sardinella-aurita-AJG36172.1 Sardinella-aurita-ADJ36462.1 Sardinella-aurita-BCK59691.1 Sardinella-aurita-ARK08442.1 Sardinella-aurita-ALF62772.1 Sardinella-aurita-ARX77980.1 Sardinella-aurita-QWC70996.1 Sardinella-aurita-CAP45731.1 Sardinella-aurita-QIS89269.1 Sardinella-aurita-QIS89268.1 Sardinella-aurita-AKI88980.1 Sardinella-aurita-ARX77952.1 Sardinella-brasiliensis-CAP45732.1 Sardinella-lemuru-QIU80653.1 Sardinella-lemuru-QIU80654.1 Sardinella-lemuru-AVR48811.1 Sardinella-lemuru-ANN81745.1
Group[2] n: 6 ;id: Sardinella-jussieu-AGD80580.1 Sardinella-albella-UAX63140.1 Sardinella-fimbriata-ADK49636.1 Sardinella-jussieu-QPT74108.1 Sardinella-goni-QNM64051.1 Sardinella-goni-QNM64052.1
Group[3] n: 2 ;id: Sardinella-maderensis-ARK08446.1 Sardinella-maderensis-YP_001293790.1
Group[4] n: 3 ;id: Sardinella-melanura-QNE86072.1 Sardinella-fimbriata-ADO15485.1 Sardinella-melanura-BBK60999.1
Group[5] n: 6 ;id: Sardinella-albella-YP_005089302.1 Sardinella-gibbosa-QLM02870.1 Sardinella-gibbosa-QOR33582.1 Sardinella-jussieu-QIX12288.1 Sardinella-sindensis-AZJ16169.1 Sardinella-sindensis-AZJ16172.1
Group[6] n: 4 ;id: Sardinella-hualiensis-QQL03349.1 Sardinella-hualiensis-QQL03348.1 Sardinella-tawilis-ADO15497.1 Sardinella-tawilis-ADO15499.1
Group[7] n: 2 ;id: Sardinella-marquesensis-AIG24222.1 Sardinella-marquesensis-AIG24223.1
Group[8] n: 2 ;id: Sardinella-zunasi-ANN81739.1 Sardinella-zunasi-ANN81740.1
Group[9] n: 1 ;id: Chirocentrus-dorab-AHY80612.1

GMYC Results

The *Sardinella* species analyzed here comprise 10 maximum likelihood entities applying the COI gene, i.e. 10 hypothetical lineages, and 6 entities applying the 12S gene (Figure S7 and Figure S8). The number of maximum likelihood clusters was 9 for COI and 5 for 12S (no. of clusters = no. of entities, except for those composed of only 1 individual, which in this case was one). The analysis using CO1 sequences was significant (p-value = 0.001, with $P < 0.05$ indicating that the null hypothesis of that is only one species is rejected). However, the analysis using the 12S sequences was not significant (p-value = 0.158), indicating that the null hypothesis of that is only one species is accepted. The lack of significance of the 12S analysis can be due to the low samples number available, which show the need of more work increasing the current database of genetic resources for fish species.

(a)

```
> summary(result)
Result of GMYC species delimitation

        method: single
        likelihood of null model:       315.1952
        maximum likelihood of GMYC model:       322.0335
        likelihood ratio:       13.67659
        result of LR test:      0.001071932**

        number of ML clusters:  9
        confidence interval:    3-10

        number of ML entities:  10
        confidence interval:    4-14

        threshold time: -0.02393692
```

(b)

```
> summary(result)
Result of GMYC species delimitation

        method: single
        likelihood of null model:       289.3994
        maximum likelihood of GMYC model:       291.2384
        likelihood ratio:       3.677925
        result of LR test:      0.1589823n.s.

        number of ML clusters:  5
        confidence interval:    1-15

        number of ML entities:  6
        confidence interval:    1-39

        threshold time: -0.009465181
```

Figure S7. Summary of the GMYC species delimitation for (a) CO1 and (b) 12S sequences of Sardinella.

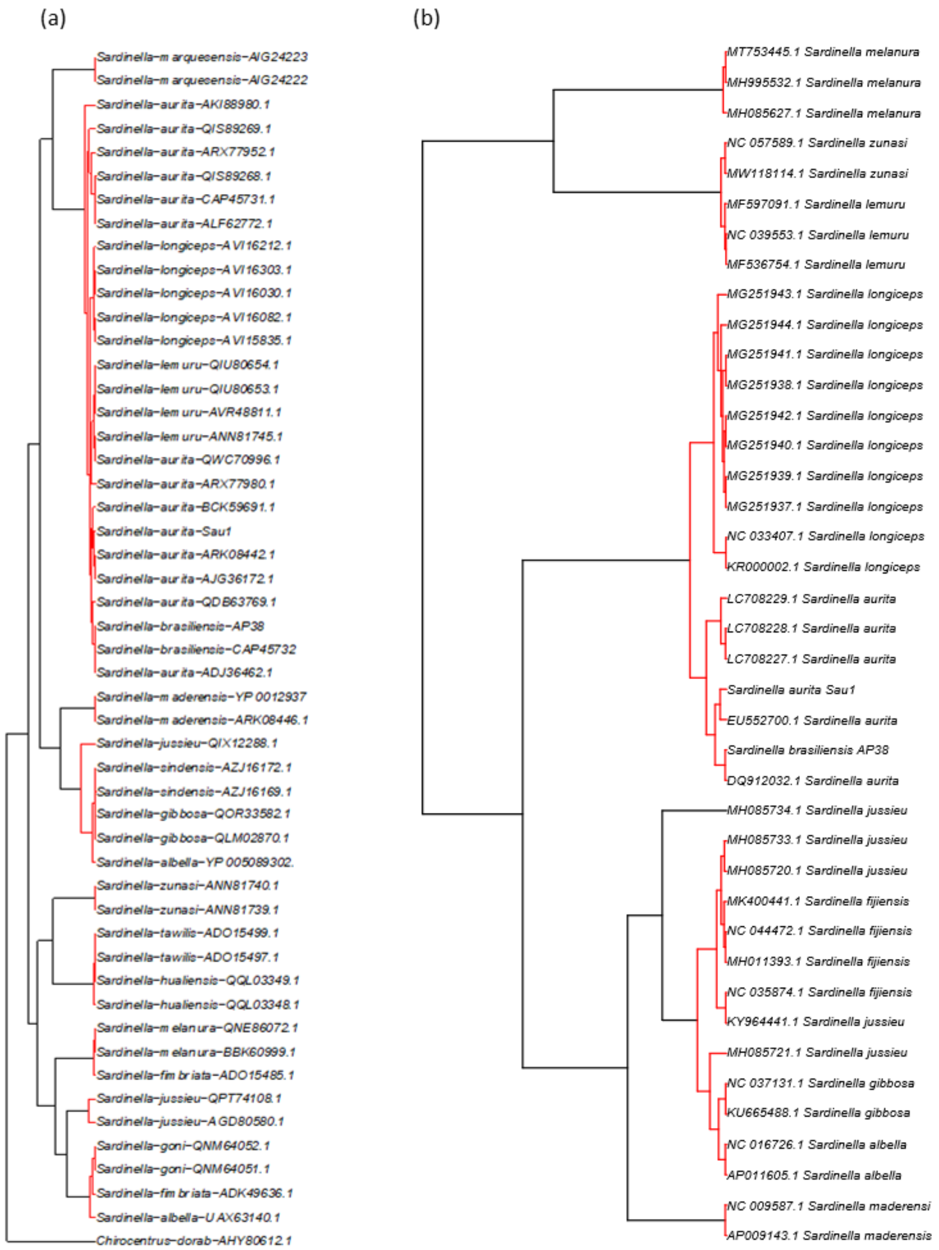

Figure S8 Lineages delimited in the GMYC analyses of the (a) COI and (b) 12S genes.

Table S7. Lineages delimited by GMYC analyses of Sardinella CO1 sequences, including the individuals that compose each one.

GMYC_spec	Lineage	sample_name
1	1	Sardinella-albella-UAX63140.1
2	1	Sardinella-fimbriata-ADK49636.1
3	1	Sardinella-goni-QNM64051.1
4	1	Sardinella-goni-QNM64052.1
5	2	Sardinella-jussieu-AGD80580.1
6	2	Sardinella-jussieu-QPT74108.1
7	3	Sardinella-fimbriata-ADO15485.1
8	3	Sardinella-melanura-BBK60999.1
9	3	Sardinella-melanura-QNE86072.1
10	4	Sardinella-hualiensis-QQL03348.1

11	4	Sardinella-hualiensis-QQL03349.1
12	4	Sardinella-tawilis-ADO15497.1
13	4	Sardinella-tawilis-ADO15499.1
14	5	Sardinella-zunasi-ANN81739.1
15	5	Sardinella-zunasi-ANN81740.1
16	6	Sardinella-albella-YP_005089302.
17	6	Sardinella-gibbosa-QLM02870.1
18	6	Sardinella-gibbosa-QOR33582.1
19	6	Sardinella-sindensis-AZJ16169.1
20	6	Sardinella-sindensis-AZJ16172.1
21	6	Sardinella-jussieu-QIX12288.1
22	7	Sardinella-maderensis-ARK08446.1
23	7	Sardinella-maderensis-YP_0012937
24	8	Sardinella-aurita-ADJ36462.1
25	8	Sardinella-brasiliensis-CAP45732
26	8	Sardinella-brasiliensis-AP38
27	8	Sardinella-aurita-QDB63769.1
28	8	Sardinella-aurita-AJG36172.1
29	8	Sardinella-aurita-ARK08442.1
30	8	Sardinella-aurita-Sau1
31	8	Sardinella-aurita-BCK59691.1
32	8	Sardinella-aurita-ARX77980.1
33	8	Sardinella-aurita-QWC70996.1
34	8	Sardinella-lemuru-ANN81745.1
35	8	Sardinella-lemuru-AVR48811.1
36	8	Sardinella-lemuru-QIU80653.1
37	8	Sardinella-lemuru-QIU80654.1
38	8	Sardinella-longiceps-AVI15835.1
39	8	Sardinella-longiceps-AVI16082.1
40	8	Sardinella-longiceps-AVI16030.1
41	8	Sardinella-longiceps-AVI16303.1
42	8	Sardinella-longiceps-AVI16212.1
43	8	Sardinella-aurita-ALF62772.1
44	8	Sardinella-aurita-CAP45731.1
45	8	Sardinella-aurita-QIS89268.1
46	8	Sardinella-aurita-ARX77952.1
47	8	Sardinella-aurita-QIS89269.1
48	8	Sardinella-aurita-AKI88980.1
49	9	Sardinella-marquesensis-AIG24222
50	9	Sardinella-marquesensis-AIG24223
51	10	Chirocentrus-dorab-AHY80612.1

Table S8. Lineages delimited by GMYC analyses of Sardinella 12S sequences, including the individuals that compose each one.

GMYC_spec	Lineage	sample_name
1	1	AP009143.1_Sardinella_maderensis
2	1	NC_009587.1_Sardinella_maderensi
3	2	AP011605.1_Sardinella_albella
4	2	NC_016726.1_Sardinella_albella
5	2	KU665488.1_Sardinella_gibbosa
6	2	NC_037131.1_Sardinella_gibbosa
7	2	MH085721.1_Sardinella_jussieu

8	2	KY964441.1_Sardinella_jussieu
9	2	NC_035874.1_Sardinella_fijiensis
10	2	MH011393.1_Sardinella_fijiensis
11	2	NC_044472.1_Sardinella_fijiensis
12	2	MK400441.1_Sardinella_fijiensis
13	2	MH085720.1_Sardinella_jussieu
14	2	MH085733.1_Sardinella_jussieu
15	3	DQ912032.1_Sardinella_aurita
16	3	Sardinella_brasiliensis_AP38
17	3	EU552700.1_Sardinella_aurita
18	3	Sardinella_aurita_Sau1
19	3	LC708227.1_Sardinella_aurita
20	3	LC708228.1_Sardinella_aurita
21	3	LC708229.1_Sardinella_aurita
22	3	KR000002.1_Sardinella_longiceps
23	3	NC_033407.1_Sardinella_longiceps
24	3	MG251937.1_Sardinella_longiceps
25	3	MG251939.1_Sardinella_longiceps
26	3	MG251940.1_Sardinella_longiceps
27	3	MG251942.1_Sardinella_longiceps
28	3	MG251938.1_Sardinella_longiceps
29	3	MG251941.1_Sardinella_longiceps
30	3	MG251944.1_Sardinella_longiceps
31	3	MG251943.1_Sardinella_longiceps
32	4	MF536754.1_Sardinella_lemuru
33	4	NC_039553.1_Sardinella_lemuru
34	4	MF597091.1_Sardinella_lemuru
35	4	MW118114.1_Sardinella_zunasi
36	4	NC_057589.1_Sardinella_zunasi
37	5	MH085627.1_Sardinella_melanura
38	5	MH995532.1_Sardinella_melanura
39	5	MT753445.1_Sardinella_melanura
40	6	MH085734.1_Sardinella_jussieu

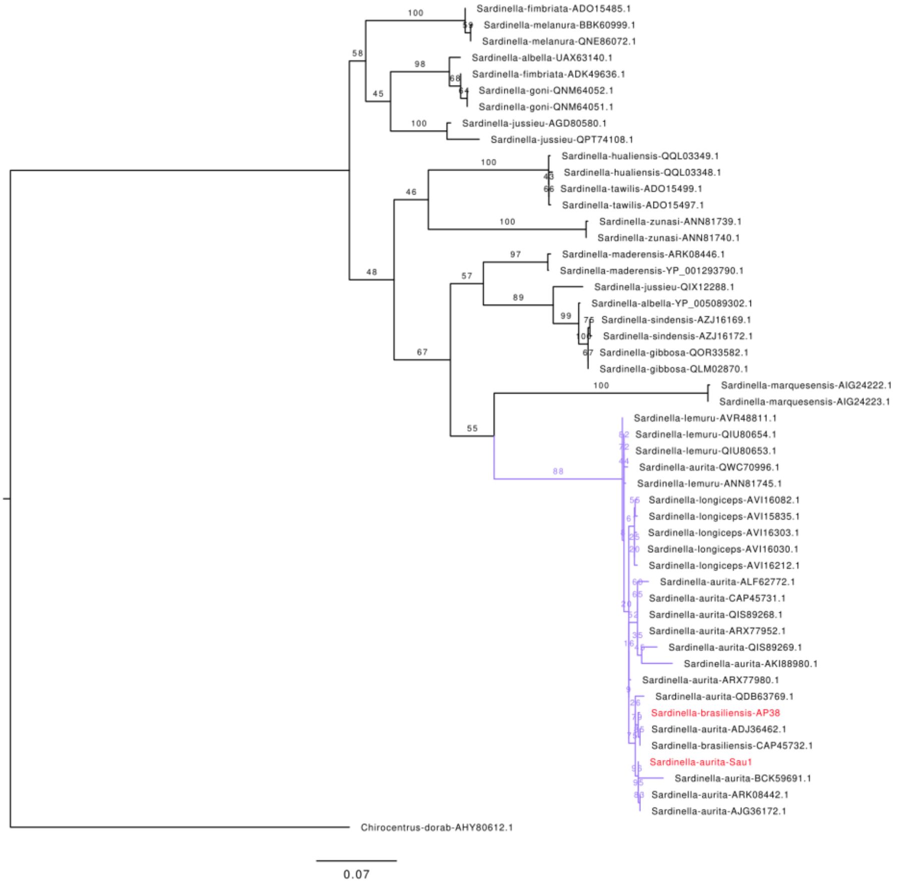

Figure S9. Phylogenetic inference of the *Sardinella* Cytochrome oxidase I (COI) sequences available in GenBank database and the ones captured here, in red. The *Sardinella Sardinella* subgenus is highlighted in purple color.

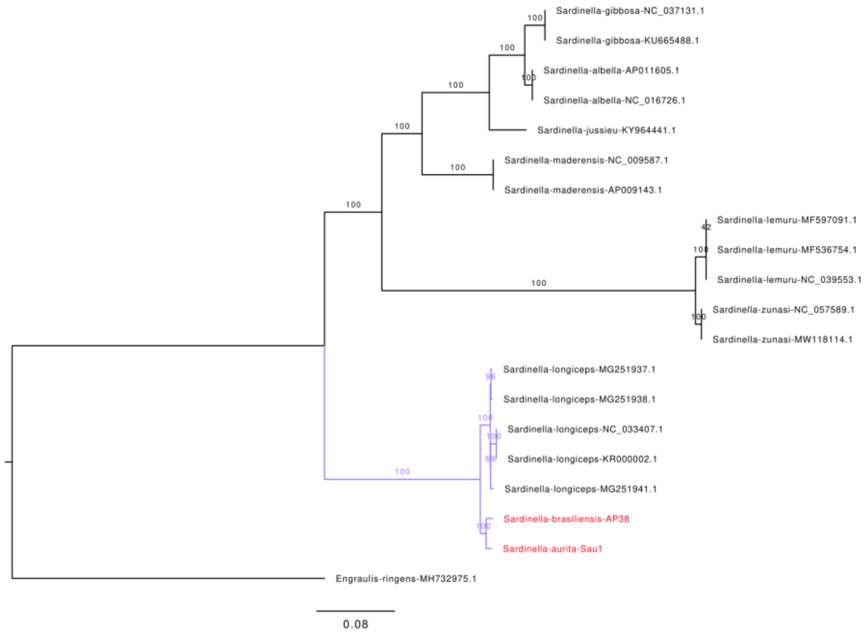

Figure S10. Phylogenetic inference of the Sardinella mitochondrial genomes available in GenBank database and the ones captured here, in red. The Sardinella Sardinella subgenus is highlighted with purple color.

PHYLOGENETIC IMPLICATIONS

Our phylogenetic inference of the complete mitochondrial genomes grouped the species correctly in their taxonomic and evolutionary positions. The phylogenetic trees could support the monophyly of 11 orders and 36 families assembled here. Perciformes, Acanthuriformes and Tetraodontiformes were split as polyphyletic orders. Two families of the order Beloniformes (Hemiramphidae and Belonidae) were identified as polyphyletic and included sequences obtained in this study. The Lotidae family was also recovered as polyphiletic, but only included sequences retrieved from GenBank.

We did not consider the polyphlyletic structure of Perciformes, Tetraodontiformes and Acanthuriformes as a methodological error in our analysis, in light of previous research that have likewise highlighted their inability to recover monophyly or their controversial taxonomic status. The two orders belong to the series Eupercaria, the largest series of percomorphs, being among the most diverse of the series [16]. Perciformes is the largest order in Eupercaria and is polyphyletic, according to the Eschmeyer's Catalog of Fishes updated classification, with 19 families classified as *sedis mutabilis*, which means that the classification may change with further studies and accurate data (Fricke et al., 2022). However, studies applying a large multilocus nuclear dataset is recovering the order as monophyletic [17, 18] and the current status of the order remain controversial. The interrelationships in Tetraodontiformes lineages are also known to be controversial, but the order is classified as monophyletic in strongly supported works (Betancur-R et al., 2017; Near et al., 2013). However, the polyphyly seen in this study for mitochondrial genome is also supported in previous research, since not only mitochondrial genes has been failing in recover monophyly in the order, but the whole mitochondrial genome itself [19, 20]. The monophyly of Acanthuriformes has also been questioned before, both by studies applying multilocus datasets associated with morphological synapomorphies (Betancur-R et al., 2017) or analyzing sequences from only five nuclear genes [21]. We therefore found evidence based on whole mitochondrial genomes that corroborate with previous research.

The recovery of the Hemiramphidae and Belonidae families as non-monophyletic groups was also not considered as an error in our analysis. The needlefishes (Belonidae) and the sauries (Scomberesocidae) form the Scomberesocoidea superfamily [22, 23], while the superfamily Exocoetoidea includes the flyingfishes (Exocoetidae), halfbeaks (Hemiramphidae) and viviparous halfbeaks (Zenarchopteridae) [24]. However, the monophyly of needlefishes (Belonidae) and halfbeaks (Hemiramphidae) has been previously refuted [25], based on a molecular phylogeny of 54 beloniform species recovered using fragments of two nuclear and two mitochondrial genes. In this topology, the observed polyphyly of Belonidae occurred due to the inclusion of sauries, which belong to the family Scomberesocidae, and the Zenarchopteridae was recovered as sister species of needlefishes/sauries. On the other hand, the Hemiramphidae family was even more problematic, including three main clades: Zenarchopteridae, the Hemiramphus clade and the Hyporhamphus/Arrhamphus clade. Although our results also recovered the inclusion of sauries in the Belonidae family, its polyphyly was also due to the inclusion of the Zenarchopteridae clade. As for the Hemiramphidae family, it was considered non-monophyletic in our analysis due to the clustering of species belonging to the Exocoetidae family. Despite some incongruences found between our results and the ones found by Lovejoy et al. (2004), it is likely that some beloniform families still lack a well-established taxonomic framework to support its monophyly. Our phylogenetic inference also sheds light into the taxonomic classification of *Sardinella* species, as discussed on the next section.

The phylogenetic inference of the COI gene also raises an important taxonomic discussion in the Mullidae family. The Argentine goatfish (*Mullus argentinae*) is a demersal fish with a narrow distribution from Argentine to northeastern Brazil (Fricke et al., 2022). Here, our *M. argentinae* collected in Southeastern Brazil (São Paulo state) clustered with the individuals collected in Argentina, but not with other individuals from the Southern Brazil (Santa Catarina state) (Figure S11), which may be an indication that the Argentine goatfish comprises cryptic species.

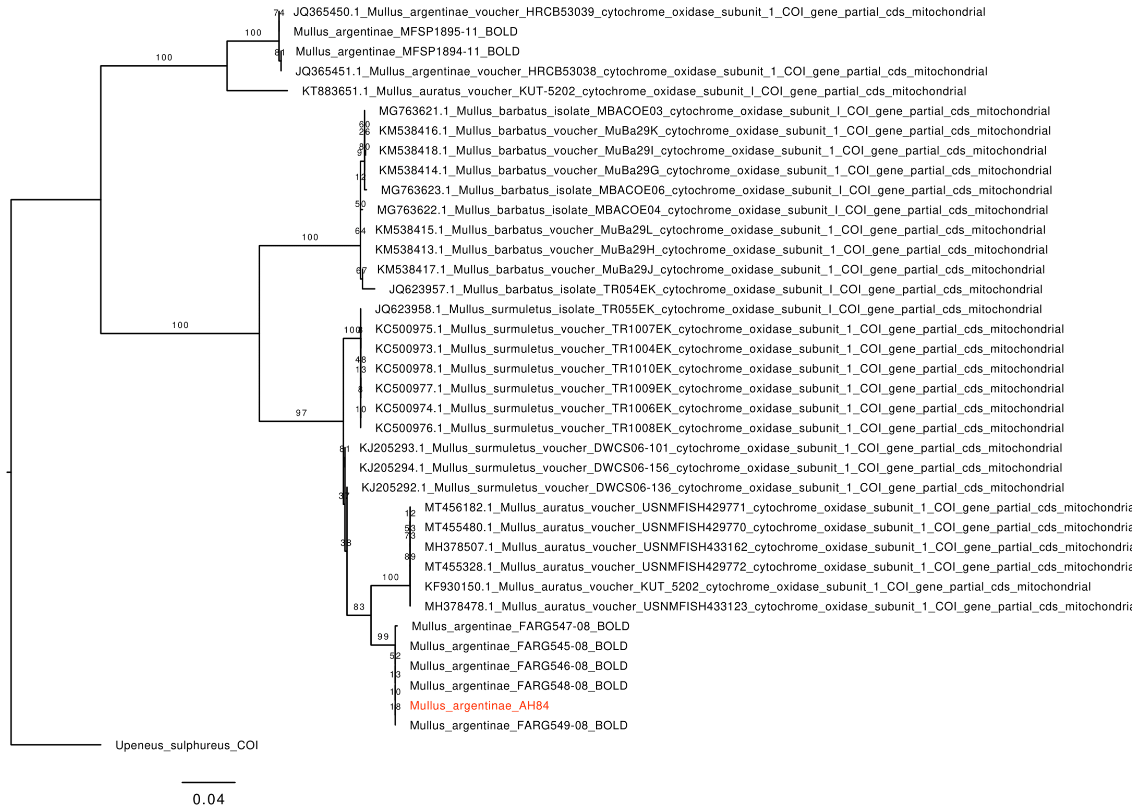

Figure S11. Phylogenetic inference of the *Mullus* Cytochrome oxidase I (COI) gene sequences available in GenBank and BOLD databases and the one captured here, in red. From the 7 sequences of the species *Mullus argentinae* available in BOLD database, 5 of them were obtained from samples collected in Argentina (FARG547-08, FARG545-08, FARG546-08, FARG548-08 and FARG549-08), and are clustered with the sequence captured here, obtained from a sample collected in southern Brazil (state of Santa Catarina). The other 2 BOLD sequences of *M. argentinae* (MFSP1895-11 and MFSP1984-11) were obtained from samples collected in southeast Brazil (state of São Paulo).

REFERENCES

1. Eschmeyer's catalog of fishes: Genera, species, references [http://researcharchive.calacademy.org/research/ichthyology/catalog/fishcatmain.asp]
2. Sayers EW, Bolton EE, Brister JR, Canese K, Chan J, Comeau DC, Farrell CM, Feldgarden M, Fine AM, Funk K *et al*. Database resources of the National Center for Biotechnology Information in 2023. Nucleic Acids Res. 2023;51:29-38. https://doi.org/10.1093/nar/gkac1032.
3. Li H, Durbin R. Fast and accurate short read alignment with Burrows–Wheeler transform. Bioinformatics. 2009;25(14):1754-1760. https://doi.org/10.1093/bioinformatics/btp324.
4. Li H, Handsaker B, Wysoker A, Fennell T, Ruan J, Homer N, Marth G, Abecasis G, Durbin R, Genome Project Data Processing S. The sequence alignment/map format and SAMtools. Bioinformatics. 2009;25(16):2078-2079. https://doi.org/10.1093/bioinformatics/btp352.
5. Walker BJ, Abeel T, Shea T, Priest M, Abouelliel A, Sakthikumar S, Cuomo CA, Zeng Q, Wortman J, Young SK *et al*. Pilon: An integrated tool for comprehensive microbial variant detection and genome assembly improvement. PLoS One. 2014;9(11):e112963. https://doi.org/10.1371/journal.pone.0112963.
6. Kozlov AM, Darriba D, Flouri T, Morel B, Stamatakis A. RAxML-NG: A fast, scalable and user-friendly tool for maximum likelihood phylogenetic inference. Bioinformatics. 2019;35(21):4453-4455. https://doi.org/10.1093/bioinformatics/btz305.

7. Kumar S, Stecher G, Li M, Knyaz C, Tamura K. MEGA X: Molecular evolutionary genetics analysis across computing platforms. Mol Biol Evol. 2018;35(6):1547-1549. https://doi.org/10.1093/molbev/msy096.
8. Schwarz G. Estimating the dimension of a model. Ann Stat. 1978;6(2):461-464.
9. Pons J, Barraclough TG, Gomez-Zurita J, Cardoso A, Duran DP, Hazell S, Kamoun S, Sumlin WD, Vogler AP. Sequence-based species delimitation for the DNA taxonomy of undescribed insects. Syst Biol. 2006;55(4):595-609. https://doi.org/10.1080/10635150600852011.
10. Ezard T, Fujisawa T, Barraclough TG. Splits: species' limits by threshold statistics. R package version. 2009;1(r29). https://doi.org/https://rdrr.io/rforge/splits/.
11. Stern N, Douek J, Goren M, Rinkevich B. With no gap to mind: A shallow genealogy within the world's most widespread small pelagic fish. Ecography. 2018;41(3):491-504. https://doi.org/10.1111/ecog.02755.
12. Nguyen L-T, Schmidt HA, von Haeseler A, Minh BQ. IQ-TREE: A fast and effective stochastic algorithm for estimating maximum-likelihood phylogenies. Mol Biol Evol. 2014;32(1):268-274. https://doi.org/10.1093/molbev/msu300.
13. Puillandre N, Lambert A, Brouillet S, Achaz G. ABGD, Automatic Barcode Gap Discovery for primary species delimitation. Mol Ecol. 2012;21(8):1864-1877. https://doi.org/10.1111/j.1365-294X.2011.05239.x.
14. Bouckaert R, Heled J, Kühnert D, Vaughan T, Wu C-H, Xie D, Suchard MA, Rambaut A, Drummond AJ. BEAST 2: A software platform for bayesian evolutionary analysis. PLoS Comp Biol. 2014;10(4):e1003537. https://doi.org/10.1371/journal.pcbi.1003537.
15. Stern N, Rinkevich B, Goren M. Integrative approach revises the frequently misidentified species of *Sardinella* (Clupeidae) of the Indo-West Pacific Ocean. J Fish Biol. 2016;89(5):2282-2305. https://doi.org/10.1111/jfb.13114.
16. Betancur-R R, Wiley EO, Arratia G, Acero A, Bailly N, Miya M, Lecointre G, Ortí G. Phylogenetic classification of bony fishes. BMC Evol Biol. 2017;17(1):162. https://doi.org/10.1186/s12862-017-0958-3.
17. Hughes LC, Ortí G, Huang Y, Sun Y, Baldwin CC, Thompson AW, Arcila D, Betancur-R R, Li C, Becker L *et al*. Comprehensive phylogeny of ray-finned fishes (Actinopterygii) based on transcriptomic and genomic data. Proceedings of the National Academy of Sciences. 2018;115(24):6249-6254. https://doi.org/10.1073/pnas.1719358115.
18. Near TJ, Eytan RI, Dornburg A, Kuhn KL, Moore JA, Davis MP, Wainwright PC, Friedman M, Smith WL. Resolution of ray-finned fish phylogeny and timing of diversification. Proceedings of the National Academy of Sciences. 2012;109(34):13698-13703. https://doi.org/10.1073/pnas.1206625109.
19. Holcroft NI. A molecular analysis of the interrelationships of tetraodontiform fishes (Acanthomorpha: Tetraodontiformes). Mol Phylogen Evol. 2005;34(3):525-544. https://doi.org/https://doi.org/10.1016/j.ympev.2004.11.003.
20. Malmstrøm M, Matschiner M, Tørresen OK, Jakobsen KS, Jentoft S. Whole genome sequencing data and de novo draft assemblies for 66 teleost species. Scientific Data. 2017;4(1):160132. https://doi.org/10.1038/sdata.2016.132.
21. Holcroft NI, Wiley EO. Acanthuroid relationships revisited: a new nuclear gene-based analysis that incorporates tetraodontiform representatives. Ichthyol Res. 2008;55(3):274-283. https://doi.org/10.1007/s10228-007-0026-x.
22. Collette BB. Beloniformes: development and relationships. Ontogeny and Systematics of Fishes. 1984.
23. Nelson JS, Grande TC, Wilson MV. Fishes of the World: John Wiley & Sons; 2016.
24. Toyama T, Kawai T, Imamura H. Phylogenetic systematics of the needlefishes (Beloniformes: Belonidae): The Thailand Natural History Museum; 2020.
25. Lovejoy NR, Iranpour M, Collette BB. Phylogeny and jaw ontogeny of beloniform fishes. Integr Comp Biol. 2004;44(5):366-377. https://doi.org/10.1093/icb/44.5.366.
